# Extended anticoagulation for the secondary prevention of venous thromboembolic events: An updated network meta-analysis

**DOI:** 10.1371/journal.pone.0214134

**Published:** 2019-04-01

**Authors:** Vicky Mai, Laurent Bertoletti, Michel Cucherat, Sabine Jardel, Claire Grange, Steeve Provencher, Jean-Christophe Lega

**Affiliations:** 1 Centre de Recherche de l’Institut Universitaire de Cardiologie et de Pneumologie de Québec, Université Laval, Ville de Québec, Canada; 2 Service de Médecine Vasculaire et Thérapeutique, CHU de St-Etienne, Saint-Etienne, France; 3 Université Jean-Monnet, UMR 1059, SAINBIOSE, St-Etienne, France; 4 INSERM CIC 1408, St-Etienne, France; 5 Groupe d’Etude Multidisciplinaire des Maladies Thrombotiques (GEMMAT), Hospices Civils de Lyon, Lyon, France; 6 Univ Lyon, UMR 5558, Laboratoire de Biométrie et Biologie Evolutive, CNRS, Villeurbanne, France; 7 Service de Pharmacologie et de toxicologie, Hospices Civils de Lyon, Lyon, France; 8 Département de Médecine Interne et Vasculaire, Centre Hospitalier Lyon Sud, Hospices Civils de Lyon, Pierre-Bénite, France; University of York, UNITED KINGDOM

## Abstract

**Background:**

Extended treatment is preconized in a significant proportion of patients with unprovoked venous thromboembolism (VTE). However, limited direct/indirect comparisons are available to appropriately weight the benefit/risk ratio of the diverse treatments available. We aimed to compare the rate of symptomatic recurrent VTE and major bleeding (MB), the net clinical benefit (VTE+MB) and death on vitamin-K antagonist (VKA), direct oral anticoagulants (DOAC) and antiplatelet drugs for extended anticoagulation.

**Methods:**

A systematic literature search through September 2018 identified randomized trials studying these pharmacologic therapies for extended anticoagulation following VTE. Treatment effects were calculated using network meta-analysis with frequentist fixed-effects model.

**Results:**

18 trials (18,221 patients) were included in the analysis. All treatments reduced the risk of recurrence compared to placebo/observation. Nonetheless, VKA (RR 0.22; 95%CI 0.13–0.39) and DOAC (RRs ranging from 0.25–0.32; 95%CI ranging from 0.13–0.52) were more effective than aspirin, whereas low-dose VKA was less effective than standard-dose VKA (RR 2.47; 95%CI 1.34–4.55). The efficacy of DOAC was globally comparable to standard-adjusted dose VKA. Low- (RR 3.13; 95%CI 1.37–7.16) and standard-dose (RR 3.23; 95%CI 1.16–8.99) VKA also increased the risk of MB, which was not the case for any DOAC. Low-dose VKA and low-dose DOAC had similar effects on MB compared to standard-doses. Although there was a trend for reduced MB and enhanced net clinical benefit for DOAC compared to VKA, this was not statistically significant. The specific anticoagulant therapies had no significant effects on deaths.

**Conclusion:**

Standard-dose VKA and low/standard-dose DOAC share similar effects on VTE recurrence and MB, whereas aspirin and low-dose VKA were associated with lower benefit/risk ratio.

## Introduction

Pulmonary embolism (PE) and deep venous thrombosis (DVT), collectively referred as venous thromboembolism (VTE), are a leading causes of death and disability worldwide, affecting 10 millions of individuals annually [1]. PE is also the third cause of mortality from cardiovascular disease, after stroke and coronary artery disease [2]. In patients with acute VTE, anticoagulant therapy markedly decreases the risk of recurrence, at the cost of an increased incidence of bleeding. This risk is varying according to the presence of underlying risk factors and thus, should obviously be balanced with the increased risk of recurrence once anticoagulant therapy is discontinued. While time-limited therapy is recommended for patients with a provoked episode of VTE, extended therapy (no scheduled stop date) is recommended after a second episode of unprovoked VTE [3]. In contrast, recent guidelines recommend a tailored anticoagulation duration according to the risk of bleeding for patients with an intermediate risk of recurrence, such as after a first episode of unprovoked VTE for which the 5-year risk of recurrence is up to 30% without anticoagulation [4, 5].

Until recently, the main option for long-term anticoagulant therapy was vitamin-K antagonists (VKA) with a standard-adjusted dose (targeting an international normalized ratio (INR) between 2 and 3) [6]. In order to decrease the risk of treatment-related bleeding, studies evaluated a lower-intensity of anticoagulant therapy with VKA (INR between 1.5 and 2), but with disappointing results both in terms of efficacy and safety [7, 8]. Other studies confirmed that aspirin (ASA) in secondary prevention of VTE may be safer, but was associated with an increased risk of recurrence [9, 10]. More recently, the development of direct oral anticoagulants (DOACs) deeply modified the landscape of anticoagulant therapy for VTE. These compounds were shown to be non-inferior in preventing recurrent VTE, with potential improvement in safety compared to standard-dose VKA during the initial phase of anticoagulation [11–13]. Their pharmacological properties also allow fixed dosages without the need for biological monitoring. Similar to VKA, reduced dosages of apixaban and rivaroxaban were recently evaluated in the extended treatment of VTE with the aim of further improving their risk/benefit ratio [13, 14].

Unfortunately, there are limited direct comparisons between treatments, and many regimens were not evaluated using the same comparator. For example, low-dose apixaban was compared to placebo [13], whereas low-dose rivaroxaban was compared to ASA [14]. The absence of direct comparisons between the various treatment regimens thus limits the clinicians’ capability to appropriately weight the risk/benefit ratio of the diverse treatments for extended anticoagulation following an acute VTE, especially for patients at intermediate risk of recurrence. We thus aimed to compare strategies based on anticoagulants and antiplatelet drugs in a network meta-analysis of trials evaluating extended anticoagulant therapy for VTE.

## Methods

This systematic review was conducted in accordance with the methodological guidelines for systematic reviews of randomized controlled trials from «Cochrane Handbook for Systematic Reviews of Interventions» [15]. No ethics approval was needed.

### Study objectives

The primary efficacy and safety objectives of the study were to compare the effects of VKA, DOAC (apixaban, dabigatran and rivaroxaban) and antiplatelet drugs for the secondary prevention of VTE on the rate of symptomatic recurrent VTE and major bleeding (MB), respectively. Secondary objectives were to assess their effects on the net clinical benefit, a composite endpoint defined as recurrent VTE or MB, as well as on fatal VTE and fatal MB.

### Data sources and searches

We updated the systematic review of Castellucci et al. [16], searching Pubmed and EMBASE using a modified search strategy up to September 30^th^ 2018 *(see online supplement)* using a filter for randomized controlled trials. Publications from potentially relevant journals were also searched by hand. There were no restrictions on language.

### Outcomes measures

The primary efficacy and safety outcome measures were recurrent VTE and MB episodes, respectively. Recurrent VTE was defined as an objectively confirmed occurrence of new DVT on ultrasound imaging, on venography or on the impedance plethysmography test, as well as a new PE suggested by a new high probability on ventilation/perfusion scanning, or a new filling defect on computed tomography or pulmonary angiography. A MB episode was defined according to International Society on Thrombosis and Haemostasis definition [17] and included fatal bleeding, bleeding in a critical area or organ, bleeding causing a fall in hemoglobin level of 20g/L or more, or leading to transfusion of two or more units of whole blood red cells. Secondary outcome measures included the net clinical benefit, as well as fatal recurrent VTE and MB episodes, defined as recurrent VTE or MB leading to death.

### Study selection, quality assessment and data extraction

Studies were independently selected and data were extracted by two reviewers (V.M. and S.J.) using a standardized data abstraction form. Studies were included in the systematic review if they met inclusion criteria defined a priori: 1) prospective enrolment of consecutive patients previously treated for a minimum of three months with anticoagulant treatment for an objectively confirmed, symptomatic DVT or PE; 2) patients randomized to receive an antiplatelet drug, a VKA or a DOAC versus placebo or observation; 3) report one of the outcomes of interest of the present systematic review. Studies recruiting patients with asymptomatic VTE were excluded. Studies’ methodological quality was assessed using the risk of bias assessment tool from the Cochrane Handbook for randomized trials [18]. The reviewers assigned a low, high or unknown risk of bias for each category. A study was considered to have a high risk of bias or an unknown risk of bias if at least one category was with a high risk or an unknown risk of bias, respectively. Primary analyses were made on all retrieved studies. The two reviewers independently extracted information from all studies retained in the meta-analysis, including 1) the study design, 2) patient characteristics, 3) mean treatment effect on VTE and MB. Two by two tables were constructed based on treatment received and available data for the primary and secondary outcomes. Only outcomes occurring during the time period that patients were receiving study drugs, placebo, or observation were included in the analysis. Disagreements were resolved by consensus.

### Statistical analysis

Frequentist network meta-analysis and direct pairwise meta-analysis were conducted for all outcomes to compute relative risk (RR) and their 95% confidence interval (95%CI). Network meta-analysis combine direct (pairwise) and indirect comparisons for the same outcome, allowing estimation of the relative effectiveness among all interventions and rank ordering of the interventions. For a given comparison (e.g. VKA versus placebo), direct evidence is provided by trials that compare these drugs directly. Indirect evidence for VKA versus placebo can be provided by synthetizing studies that compared VKA versus aspirin and placebo versus aspirin. Network meta-analysis combines both direct and indirect evidence across a network of studies into a single effect size for a given medical condition. This method is similar to electrical network, where variance corresponds to resistance, treatment to voltage, and weighted treatment effects to current flows [19]. We assessed available studies and patient characteristics to ensure similarity and to investigate the potential effect of heterogeneity on effect estimates. Placebo and observation were combined within the evidence network [16]. We used adjusted continuity corrections of 0.5 to studies with no event [20]. Comparisons with zero events in each study arms were not considered in network meta-analysis. I^2^ statistic was used to assess between study heterogeneity and was considered high at I^2^>50%. Fixed-effect model was used in regard to negligible or moderate heterogeneity (i.e. I^2^ <50% for all outcomes, range 0–24%). We then calculated the probability that each drug had the most efficacious regimen by the p-score, which can be considered as a frequentist analogue of the surface under the cumulative ranking curve for Bayesian approach [21].

We systematically tested the presence of significant interaction between the estimate of treatment effects derived from direct and indirect meta-analysis. A sensitivity analysis was planned a priori, adding drugs are not commercialized for the treatment of VTE (ximelagatran, idraparinux and sulodexide) from the evidence network. The pooled prevalence of events and its 95%CI was estimated using the arcsine transformation. All analyses were performed using R (netmeta package version 0.9–5 for treatment comparison, meta package version 4.8–2 for pooled prevalence, R Language and Environment for Statistical Computing, Vienna, Austria).

## Results

### Study selection and characteristics of included randomized controlled trials

**Fig 1:**
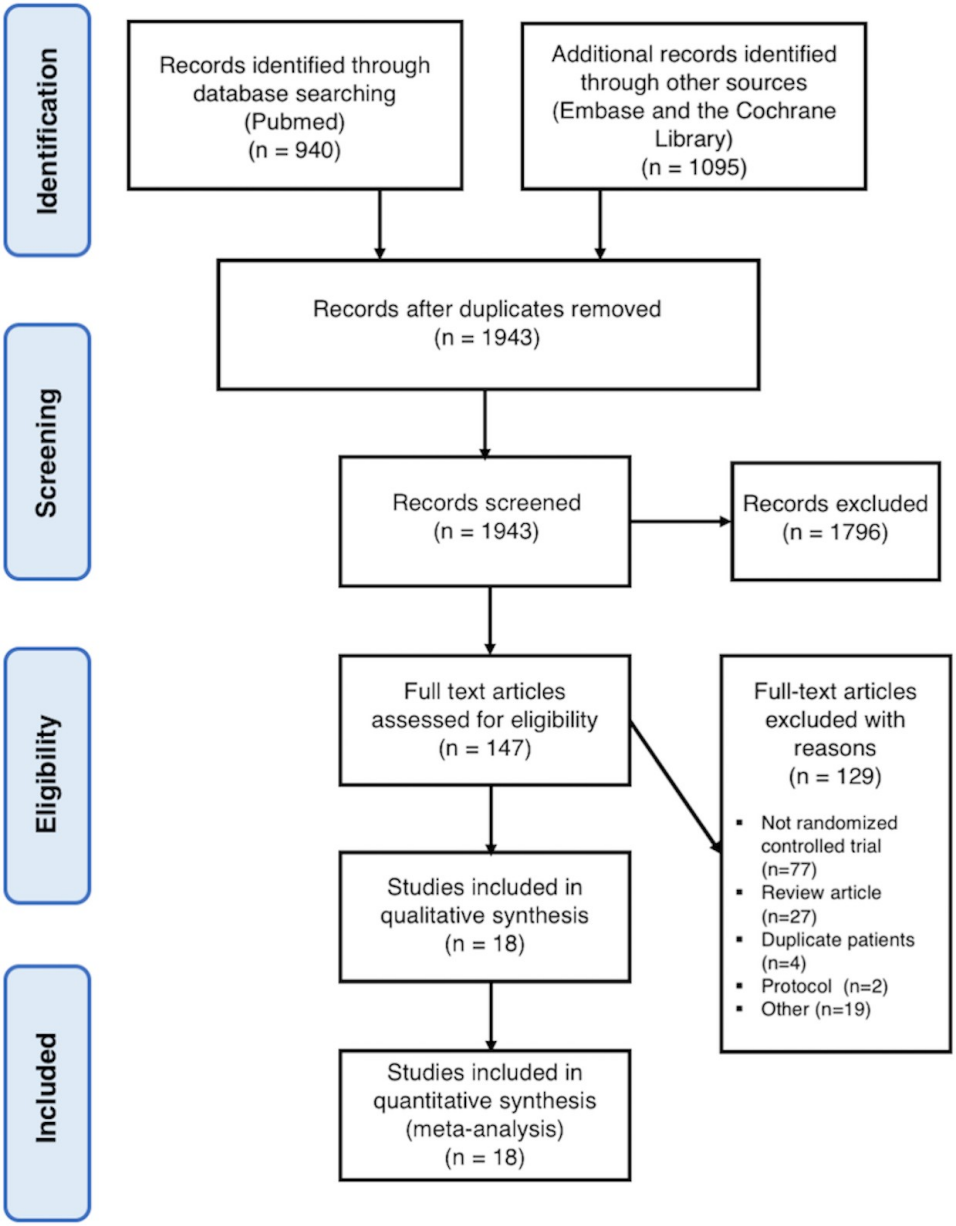
Study flow chart.

**Fig 2:**
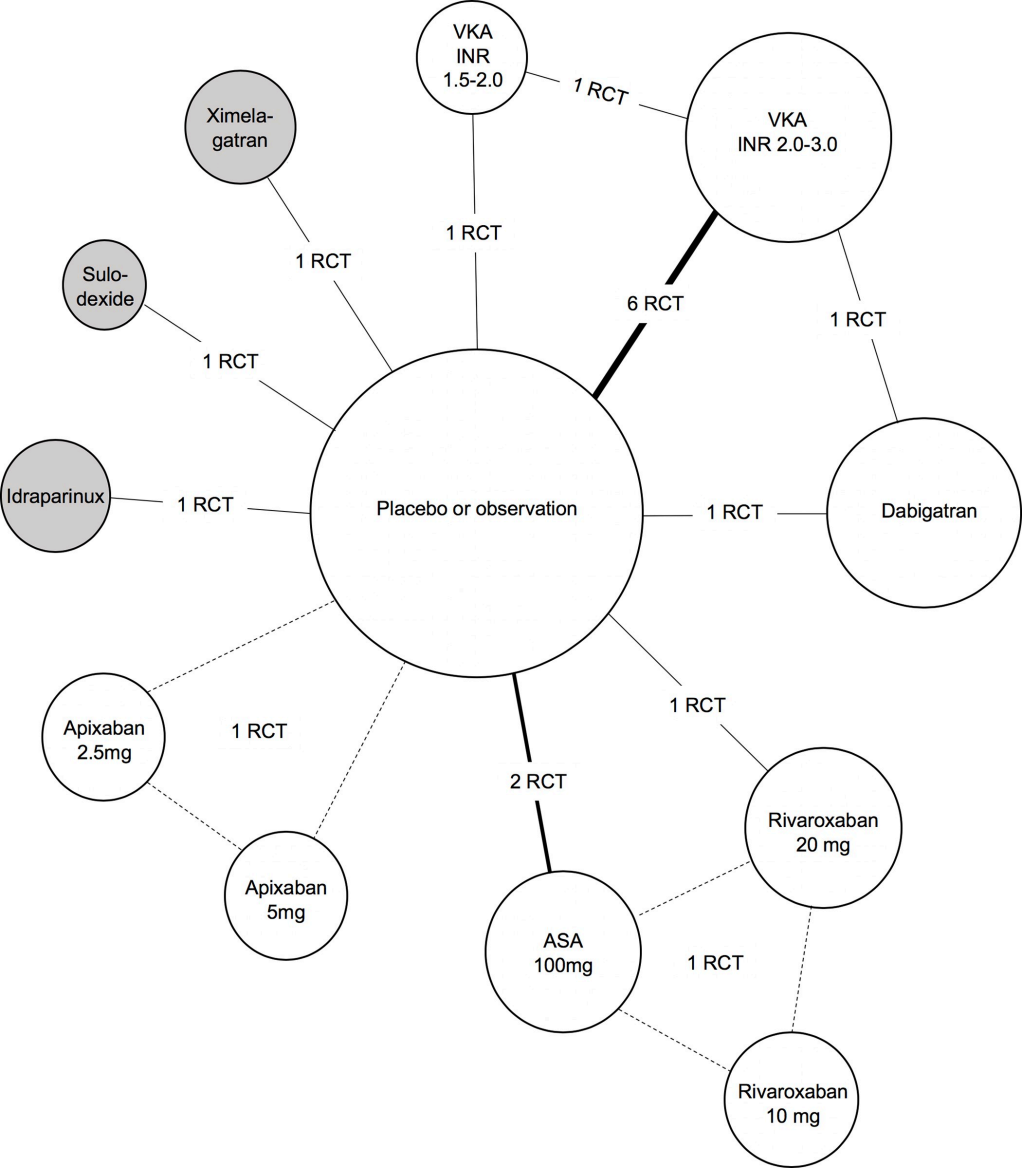
Evidence network of included studies. The width of lines for each connection in the evidence network are proportional to the number of randomized controlled trials (RCTs) comparing each pair of treatments. Multiarm trials are represented in dotted lines. The size of each treatment node is proportional to the number of randomized participants (sample size). Direct oral anticoagulants for the main analysis included apixaban, dabigatran and rivaroxaban, whereas unmarketed drugs (shaded in gray) **(**idraparinux, sulodexide and ximelagatran) were used for sensitivity analyses only (see online supplement). ASA: aspirin; RCT: randomized controlled trial; VKA: vitamin K antagonist.

The primary reviewers included 18 independent trials that contributed to 17 separate publications [7–9, 11–14, 22–31], representing 18,221 patients (median sample size of 678). The reasons for excluding studies appear in **Fig 1**. Patients and study characteristics are shown in **Table 1**. Number of events for each main outcome are reported in **S1 Table**. Ten studies recruited patients with unprovoked VTE only [7–9, 22, 23, 25–27, 30, 31], whereas the proportion of unprovoked VTE was 64±4% (range 41–92%) and unknown in seven [11–14, 24, 29] and in one [28] study, respectively. Acute PE represented 33±31% (range 0 to 100%) of index events. Underlying cancers were unusual. Trials assessed the effects of VKA (n = 8) [7, 8, 22–27], DOAC (n = 6) [11–14, 28], ASA (n = 2) [9, 30], idraparinux (n = 1) [29] and sulodexide (n = 1) [31] against standard treatments (VKA or ASA) or placebo/observation (**Fig 2**). The mean follow-up duration was 24±11 months. Overall, included trials were generally at low risk of bias (**S2 Table**). Although independent, blinded outcome assessments were described for all trials, lack of blinding was noted for four trials of them [23, 24, 26, 27], whereas allocation concealment also was not reported in one study [26].

**Table 1:** Baseline characteristics and results of included trials.

Study (Patients)	Design	Initial therapy before randomization	Interventions groups	Number of patients	Mean treatment duration (months) ^a^	Mean follow-up (months) ^a^	Mean age (years)	Men (%)	PE as index event (%)	Unprovoked VTE (%)	Cancer at randomization (%)
Kearon 1999[22] (all VTE)	Double blind, randomized	UH or LMWH, followed by VKA for 3 months	Placebo	83	24^b^^,^ ^c^	9	58	53	27	100	0^d^
			VKA (INR 2.0–3.0)	79	24^b^^,^ ^c^	12	59	68	24	100	0^d^
Agnelli 2001[23] (DVT only)	Open label, randomized	UH or LMWH, followed by VKA for 3 months	Observation	133	9	37	68	61	0	100	0^e^
			VKA (INR 2.0–3.0)	134	9	38	67	55	0	100	0^e^
Agnelli 2003[24] (PE only)	Open label, randomized	VKA for 3 months	Observation	161	3 vs 9^f^	33	61	42	100	57	0^e^
			VKA (INR 2.0–3.0)	165	3 vs 9^f^	35	63	39	100	56	0^e^
Couturaud 2015[25] (PE only)	Double blind, randomized	VKA for 6 months	Placebo	187	18	23^g^	57	55	100	100	3.2^h^
			VKA (INR 2.0–3.0)	184	18	23^g^	59	42	100	100	4.3^h^
Eischer 2009[26] (all VTE)^i^	Open label, randomized	UH or LMWH, followed by VKA for 6 months	Observation	17	24	37^b^^,^ ^j^	54	35	35	100	0^e^
			VKA (INR 2.0–3.0)	17	24	37^b^^,^ ^j^	53	29	47	100	0^e^
Palareti 2006[27] (all VTE)^k^	Open label, randomized	VKA	Observation	122^l^	18	17	68	42	39	100	0^e^
			VKA (INR 2.0–3.0)	105^l^	18	17	70	53	35	100	0^e^
Kearon 2003[7] (all VTE)	Double blind, randomized	VKA for 3 months	VKA (INR 2.0–3.0)	369	26	29	57	53	38	100	0^m^
			VKA (INR 1.5–1.9)	369	25	29	57	57	32	100	0^m^
Ridker 2003[8] (all VTE)	Double blind, randomized	VKA for 3 months	Placebo	253	25^n^	25	53^g^^,^ ^o^	53	NR	100	NR^p^
			VKA (INR 1.5–2.0)	255	25^n^	25	53^g^^,^ ^o^	53	NR	100	NR^p^
Schulman 2003[28] (all VTE)	Double blind, randomized	Anticoagulant therapy for 6 months	Placebo	616^q^	17	19^b^	58	51	36	NR	5
			Ximelagatran 24mg BID	617^q^	17	19^b^	56	54	33	NR	6
Schulman 2013[11] (all VTE)	Double blind, randomized	Approved anticoagulant or dabigatran^r^ for 6–18 months	Placebo	668^s^	6	18^b^	56	55	32	84^t^	0.3^u^^,^ ^v^
			Dabigatran 150mg BID	685^s^	6	18^b^	56	56	34	79^t^	0.1^u^^,^^v^
Schulman 2013[11] (all VTE)	Double blind, randomized	Approved anticoagulant or dabigatran^r^ for 3–12 months	VKA (INR 2.0–3.0)	1431^w^	18^b^^,^ ^x^	36^b^	54	61	35	70	4.1
			Dabigatran 150mg BID	1435^w^	18^b^^,^ ^x^	36^b^	55	61	34	71	4.2
Einstein Investigators 2010[12] (all VTE)	Double blind, randomized	VKA or rivaroxaban^y^ for 6–12 months	Placebo	595^z^	6 or 12	7 or 13^b^	58	57	40	74	4.4
			Rivaroxaban 20mg DIE	602^z^	6 or 12	7 or 13^b^	58	59	36	73	4.7
Weitz 2017[14] (all VTE)	Double blind, randomized	VKA, dabigatran, rivaroxaban, apixaban or edoxaban for 6–12 months	ASA 100mg DIE	1139^aa^	12^g^	13^b^	59	57	48	41	3.3
			Rivaroxaban 10 mg DIE	1136^aa^	12^g^	13^b^	59	55	50	43	2.4
			Rivaroxaban 20 mg DIE	1121^aa^	12^g^	13^b^	58	54	48	40	2.3
Agnelli 2013[13] (all VTE)	Double blind, randomized	VKA, apixaban, enoxaparin or warfarin^ab^ for 6–12 months	Placebo	829^ac^	12	13^b^	57	57	34	91	2.2
			Apixaban 5mg BID	815^ac^	12	13^b^	56	58	35	91	1.1
			Apixaban 2.5mg BID	842^ac^	12	13^b^	57	58	35	93	1.8
Van Gogh 2007[29] (all VTE)	Double blind, randomized	VKA or idraparinux^ad^ for 6 months	Placebo	621^ae^	6	9 to 12^b^^,^ ^af^	60	53	49	60	10.8
			Idraparinux 2.5mg s/c once weekly	594^ae^	6	9 to 12^b^^,^ ^af^	60	53	48	61	8.9
Becattini 2012[30] (all VTE)	Double blind, randomized	VKA for 6–18 months	Placebo	198^ag^	24^g^	24^g^	62	62	34	100	NR^e^
			ASA 100mg DIE	205^ag^	24^g^	25^g^	62	66	41	100	NR^e^
Brighton 2012[9] (all VTE)	Double blind, randomized	Heparin followed by VKA (or an effective alternative anticoagulant) for 1.5–24 months	Placebo	411	27^ah^	37^g^	54	54	43	100^ai^	2^aj^
			ASA 100mg DIE	411	27^ah^	37^g^	55	55	41	100^ai^	2^aj^
Andreozzi 2015[31] (all VTE)	Double blind, randomized	VKA for 3–12 months	Placebo	309^ak^	24^g^	24^b^	56	50	8	100	NR^al^
			Sulodexide 500 lipasemic units BID	308^ak^	24^g^	24^b^	56	57	8	100	NR^al^

^a^Rounded up to the nearest unit

^b^Intended

^c^Actual mean duration: 10 months

^d^Excluded if cancer in the last five years

^e^Excluded if known cancer

^f^3 months for transient risk factor vs 9 months for idiopathic index event

^g^Median

^h^Previous cancer which resolved 2 years before randomization

^i^With FVIII levels >230 IU/dL

^j^Mean follow-up: 37 months, but the extracted data was up to 24 months to uniform the data

^k^Abnormal d-dimer level 1 month after discontinuation of anticoagulation (received at least 3 months of VKA as initial treatment)

^l^103 in observation group and 120 in VKA group were included in the intention-to-treat analyses

^m^Excluded if active cancer within the last 2 years

^n^6.5 months was the median

^o^Inclusion criteria included to be 30 years old and up

^p^Excluded if history of metastatic cancer

^q^5 patients in each group were excluded from the intention-to-treat analyses because no data were available for them after randomization

^r^from RE-COVER or RE-COVER II

^s^662 in placebo group and 681 in dabigatran group were included in the modified intention-to-treat analyses

^t^Previous cancer excluded (6 in each group), 2 in placebo group and 1 in dabigatran group had active cancer and were included which violated the protocol

^u^Protocol violation

^v^5.6 in placebo group and 6.5 in dabigatran group had previous cancer

^w^1426 in VKA group and 1430 in dabigatran group were included in the modified intention-to treat analyses

^x^Extension of the planned treatment, resulting in a treatment period of 6 to 36 months

^y^VKA (from EINSTEIN studies or routine care) or rivaroxaban (from EINSTEIN studies)

^z^594 in placebo group and 602 in rivaroxaban group were included in the modified intention-to-treat analyses

^aa^1131 in ASA group, 1127 in rivaroxaban 10mg group and 1107 rivaroxaban 20mg group were included in the intention-to-treat analyses, because patients who were randomized were excluded from the intention-to-treat analyses if they didn’t take any study medication

^ab^From AMPLIFY trial

^ac^829 in placebo group, 815 in apixaban 5 mg group and 842 in apixaban 2,5mg group were included in the intention-to-treat analyses

^ad^VKA (in previous Van Gogh studies or outside the studies) or idraparinux (in Van Gogh studies)

^ae^621 in placebo group and 594 in idraparinux group were included in the efficacy analyses and 616 in placebo group and 594 in idraparinux group were included in the safety analyses

^af^Data extracted during the 6 months treatment; ASA: aspirin

^ag^197 in placebo group and 205 in ASA group were included in the modified intention-to-treat analyses because they had received at least one done of the study drug

^ah^Intended treatment period: 2 to 4 years

^ai^One of the inclusion criteria of the study was to have a first unprovoked venous thromboembolism, but 5% had a previous provoked venous thromboembolic event and 2% had an active cancer

^aj^The authors said that there was 2% of active cancers but didn’t precise how many was in each group, so the numbers were extrapolated

^ak^308 in placebo group and 307 in sulodexide group were included in the efficacy analyses

^al^Excluded if solid neoplasm.

BID: twice daily; DIE: once daily; INR: international normalized ratio; LMWH: low molecular weight heparin; NR: not reported; PE: pulmonary embolism; VKA: vitamin K antagonist; VTE: venous thromboembolism; UH: unfractionated heparin.

### Recurrent venous thromboembolic events

**Fig 3:**
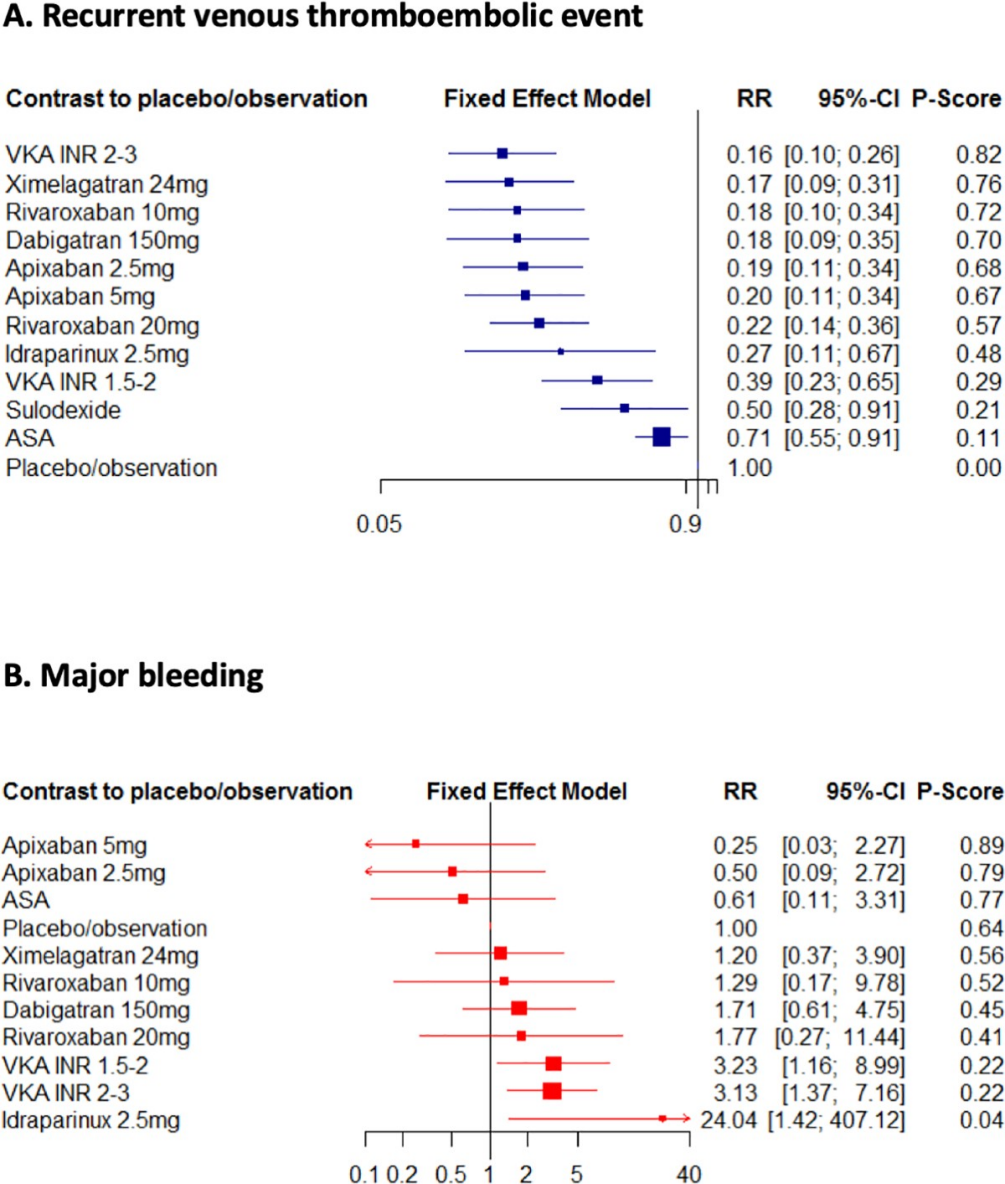
**Relative risks (95% confidence interval) for (A) recurrent venous thromboembolic events and (B) major bleeding in network meta-analysis versus observation or placebo.** ASA: aspirin; INR: international normalized ratio; VKA: vitamin k antagonist.

The analysis of VTE recurrence encompassed 17 trials (17,895 patients) that contributed to 16 publications [7–9, 11–14, 22, 23, 25–31], allowing 21 comparisons. The rate of recurrent VTE was 2.8% (95%CI 1.9–3.9%, I^2^ = 91%) and 11.2% (95%CI 8.6–14.2%, I^2^ = 89%) in patients with and without active anticoagulation, respectively (overall rate of 5.4%, 95%CI 5.4–7.1%, I^2^ = 95%). The estimates of the treatment-effect derived from the direct and indirect meta-analysis were not different. Overall, all treatments reduced the risk of recurrence compared to placebo or observation (**Fig 3A**). In multiple pairwise comparisons (**Table 2**), however, VKA and DOAC were more effective than ASA and standard-dose VKA was more effective than low-dose VKA. Standard- and low-dose DOAC also tended to be more effective than low-dose VKA, whereas their efficacy was globally comparable to standard-dose VKA. Sensitivity analysis including unmarketed drugs **(**idraparinux, sulodexide and ximelagatran) yielded similar results **(S3 Table).** Frequentist network meta-analyses suggested that standard-dose VKA was associated with the highest probability of being the best treatment for VTE reduction (82%) (**Table 3)**. Data providing estimates for unmarketed drugs are detailed in **S4 Table**.

**Table 2:** Relative risk (95% confidence interval) from network meta-analysis for recurrent thromboembolism events and major bleeding for all pairwise comparisons.

**Placebo or** **observation**	0.61 (0.11–3.31)	**3.13** **(1.37–7.16)**	**3.23** **(1.16–8.99)**	1.71 (0.61–4.75)	0.50 (0.09–2.72)	0.25 (0.03–2.27)	1.29 (0.17–9.78)	1.77 (0.27–11.44)
**0.71** **(0.55–0.91)**	**ASA**	5.16 (0.78–34.04)	5.32 (0.73–38.56)	2.81 (0.39–20.39)	0.82 (0.07–9.06)	0.42 (0.03–6.68)	2.13 (0.53–8.53)	2.92 (0.81–10.59)
**0.16** **(0.10–0.26)**	**0.22** **(0.13–0.39)**	**VKA** **(INR 2.0–3.0)**	1.03 (0.44–2.39)	0.55 (0.28–1.05)	0.16 (0.02–1.05)	**0.08** **(0.01–0.84)**	0.41 (0.05–3.68)	0.57 (0.07–4.35)
**0.39** **(0.23–0.65)**	**0.55** **(0.31–0.97)**	**2.47** **(1.34–4.55)**	**VKA** **(INR 1.5–2.0)**	0.53 (0.18–1.52)	0.15 (0.02–1.12)	**0.08** **(0.01–0.88)**	0.40 (0.04–3.87)	0.55 (0.07–4.61)
**0.18** **(0.09–0.35)**	**0.26** **(0.13–0.52)**	1.16 (0.68–1.99)	0.47 (0.22–1.02)	**Dabigatran** **150 mg BID**	0.29 (0.04–2.12)	0.15 (0.01–1.67)	0.76 (0.08–7.31)	1.04 (0.12–8.71)
**0.19** **(0.11–0.34)**	**0.27** **(0.15–0.50)**	1.22 (0.57–2.60)	0.49 (0.23–1.06)	1.03 (0.43–2.49)	**Apixaban** **2.5 mg BID**	0.51 (0.05–5.60)	2.58 (0.18–36.21)	3.54 (0.28–44.05)
**0.20** **(0.11–0.34)**	**0.28** **(0.15–0.51)**	1.24 (0.58–2.64)	0.50 (0.23–1.08)	1.05 (0.43–2.54)	1.02 (0.49–2.12)	**Apixaban** **5 mg BID**	5.08 (0.26–100.17)	6.97 (0.39–123.63)
**0.18** **(0.09–0.35)**	**0.25** **(0.14–0.46)**	1.15 (0.51–2.60)	0.47 (0.20–1.06)	0.97 (0.38–2.47)	0.94 (0.40–2.21)	0.93 (0.39–2.18)	**Rivaroxaban** **10 mg daily**	1.37 (0.42–4.43)
**0.22** **(0.14–0.36)**	**0.32** **(0.20–0.49)**	1.42 (0.72–2.83)	0.58 (0.29–1.16)	1.20 (0.53–2.75)	1.17 (0.56–2.43)	1.15 (0.55–2.39)	1.24 (0.63–2.44)	**Rivaroxaban** **20 mg daily**

Relative risks for recurrent venous thromboembolism are below the diagonal line (row defining the experimental group, column defining the placebo/observation group), whereas relative risks for major bleeding are above the diagonal line (row defining placebo/observation group, column defining the experimental group). Significant results are represented in bold/light grey. Data for non-commercialized drugs (idraparinux, sulodexide, and ximelagatran) are provided in S3 Table. ASA: aspirin; BID: twice daily; VKA: vitamin K antagonist.

**Table 3:** Probability of being the best treatment according to the p-score computing using frequentist network meta-analysis.

Treatment	Recurrence of VTE	Major bleeding	Net clinical benefit	Fatal recurrent VTE and MB
Placebo/observation	0%	77%	2%	34%
ASA 100 mg DIE	13%	75%	18%	15%
Low-dose VKA (INR 1.5–2.0)	27%	22%	30%	74%
Standard-dose VKA (INR 2.0–3.0)	**82%**	22%	54%	51%
Dabigatran 150mg BID	69%	45%	73%	72%
Apixaban 2.5 mg BID	67%	**79%**	**81%**	70%
Apixaban 5 mg BID	66%	**89%**	**84%**	59%
Rivaroxaban 10 mg DIE	71%	52%	76%	66%
Rivaroxaban 20 mg DIE	55%	41%	61%	25%

Note that in the absence of confidence intervals, these estimates should be interpreted with great caution. Data for non-commercialized drugs (idraparinux, sulodexide, and ximelagatran) are provided in S4 Table. ASA: aspirin; BID: twice daily; DIE: once daily; MB: major bleeding; VKA: vitamin K antagonist; VTE: venous thromboembolism.

### Risk of major bleeding

The analysis of MB also included 17 trials (17,604 patients) that contributed to 16 separate publications [7–9, 11–14, 22–30], allowing 21 comparisons. One study was excluded from the analysis in the absence of MB in both study arms [31]. The overall rate of MB was 0.8% (95%CI 0.5–1.2%, I^2^ = 78%) and 0.3% (95%CI 0.1–0.6%, I^2^ = 68%) in patients with and without active anticoagulation, respectively (overall rate 0.6%; 95%CI 0.4–0.9%, I^2^ = 77%). Overall, low- and standard-dose VKA and idraparinux significantly increased the risk of MB compared to placebo or observation (**Fig 3B**). DOAC tended to be associated with a decreased risk of MB compared to VKA (**Table 2**). This was statistically significant for apixaban 5mg only. Apixaban 5 mg (89%) and 2.5 mg (79%) were associated with the highest probability of being the best treatments in terms of MB risk (**Table 3**).

### Net clinical benefit and risk of fatal outcomes

**Fig 4:**
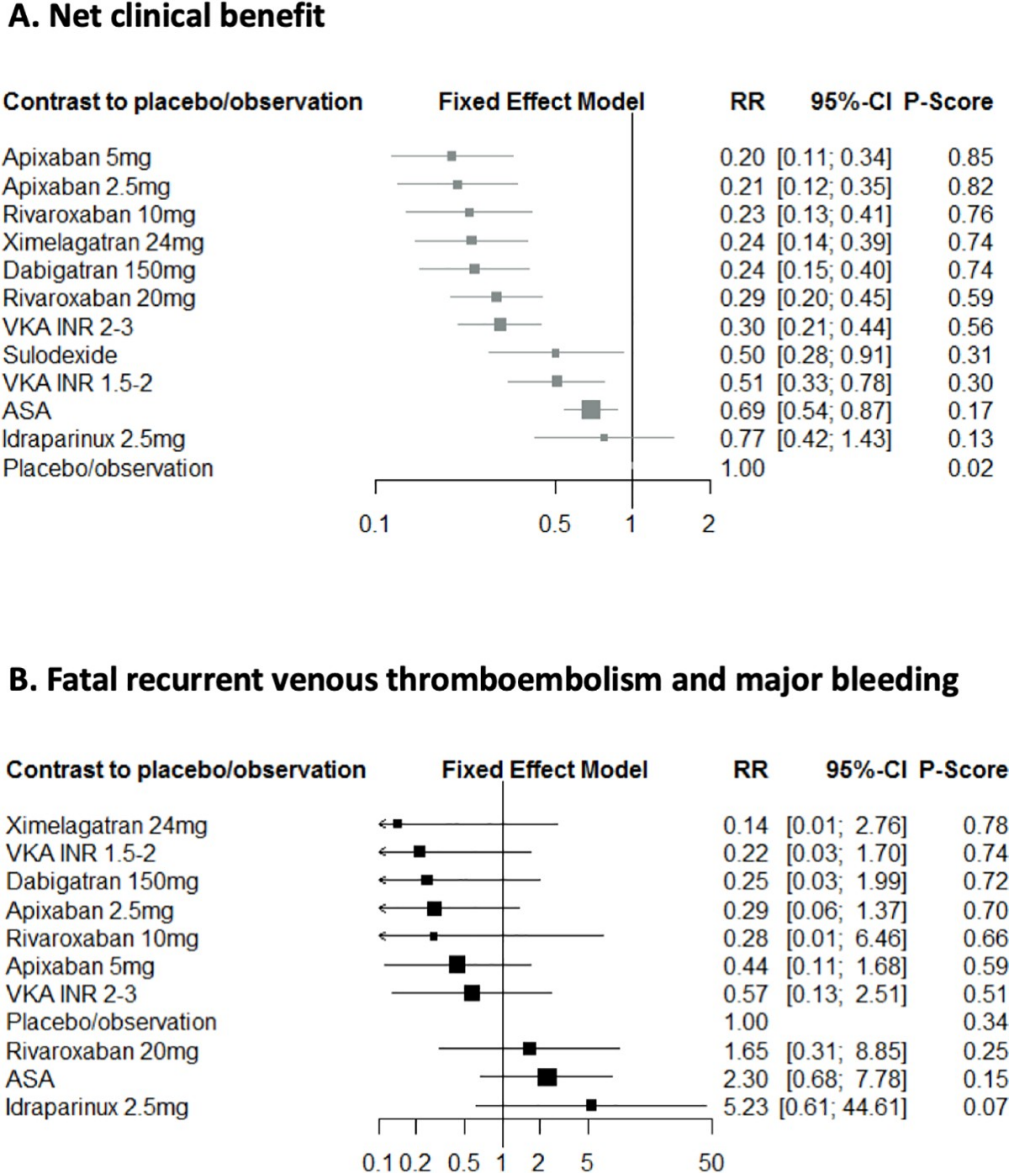
**Relative risks (95% confidence interval) for net clinical benefit (A) and death related to fatal recurrent venous thromboembolism and major bleeding (B) in network meta-analysis versus observation or placebo.** ASA: aspirin; INR: international normalized ratio; VKA: vitamin k antagonist.

The analysis of net clinical benefit included 17,895 patients from 17 independent trials that contributed to 16 separate publications [7–9, 11–14, 22, 23, 25–31], allowing 21 comparisons. All therapies were associated with significant net clinical benefit compared to placebo, except for idraparinux (**Fig 4A**). However, results from the network meta-analysis showed that standard-dose VKA and DOAC were associated with significantly higher net clinical benefit than ASA and low-dose VKA (**Table 4**, see **S5 Table** for estimates of unmarketed drugs). Apixaban 5mg (84%) and 2.5mg (81%) were also associated with the highest probability of being the best treatments for the net clinical benefit (**Table 3**). Conversely, none of the treatment was associated with a reduction in fatal outcome due to recurrent VTE or MB (**Fig 4B**) within the 13 trials (16,569 patients) [7–9, 11–14, 22–24, 28, 29], allowing 17 comparisons. In multiple pairwise comparisons, only apixaban 2.5 mg twice daily compared to ASA was associated with reduced fatal events (**Table 4**). This analysis was limited by the overall low mortality due to fatal VTE (0.18%; 95%CI 0.10–0.29%) and MB (0.02%; 95%CI 0.00–0.04%).

**Table 4:** Relative risk (95% confidence interval) from network meta-analysis for net clinical benefit and fatal recurrent venous thromboembolism and major bleeding for all pairwise comparisons.

**Placebo or** **observation**	2.30 (0.68–7.78)	0.57 (0.13–2.51)	0.22 (0.03–1.70)	0.25 (0.03–1.99)	0.29 (0.06–1.37)	0.44 (0.11–1.68)	0.28 (0.01–6.46)	1.65 (0.31–8.85)
**0.69** **(0.54–0.87)**	**ASA**	0.25 (0.04–1.69)	0.09 (0.01–1.03)	0.11 (0.01–1.20)	**0.12** **(0.02–0.91)**	0.19 (0.03–1.17)	0.12 (0.01–2.30)	0.72 (0.18–2.80)
**0.30** **(0.21–0.44)**	**0.44** **(0.29–0.69)**	**VKA** **(INR 2.0–3.0)**	0.38 (0.05–2.68)	0.43 (0.06–3.09)	0.50 (0.06–4.37)	0.77 (0.10–5.72)	0.49 (0.02–15.89)	2.92 (0.31–27.43)
**0.51** **(0.33–0.78)**	0.74 (0.45–1.21)	**1.62** **(1.01–2.58)**	**VKA** **(INR 1.5–2.0)**	1.14 (0.08–16.06)	1.33 (0.10–17.76)	2.02 (0.17–23.88)	1.29 (0.03–55.60)	7.68 (0.54-109-94)
**0.24** **(0.15–0.40)**	**0.35** **(0.20–0.61)**	0.79 (0.53–1.17)	**0.48** **(0.27–0.85)**	**Dabigatran** **150 mg BID**	1.17 (0.09–15.94)	1.78 (0.15–21.45)	1.13 (0.03–49.56)	6.75 (0.46–98.58)
**0.21** **(0.12–0.35)**	**0.30** **(0.17–0.54)**	0.65 (0.34–1.26)	**0.41** **(0.21–0.81)**	0.86 (0.42–1.77)	**Apixaban** **2.5 mg BID**	1.53 (0.26–9.11)	0.97 (0.03–32.68)	5.79 (0.58–57.51)
**0.20** **(0.11–0.34)**	**0.29** **(0.16–0.52)**	0.62 (0.32–1.21)	**0.39** **(0.20–0.78)**	0.82 (0.39–1.71)	0.95 (0.47–1.92)	**Apixaban** **5 mg BID**	0.64 (0.02–19.53)	3.79 (0.44–32.64)
**0.23** **(0.13–0.41)**	**0.34** **(0.20–0.57)**	0.73 (0.37–1.44)	**0.46** **(0.23–0.93)**	0.96 (0.45–2.03)	1.12 (0.51–2.42)	1.17 (0.53–2.56)	**Rivaroxaban** **10 mg daily**	5.97 (0.31–113.79)
**0.29** **(0.20–0.45)**	**0.43** **(0.29–0.64)**	0.92 (0.52–1.63)	0.58 (0.32–1.06)	1.22 (0.64–2.32)	1.42 (0.73–2.78)	1.49 (0.75–2.95)	1.27 (0.71–2.27)	**Rivaroxaban** **20 mg daily**

Relative risks for net clinical benefit are below the diagonal line (row defining the experimental group, column defining the placebo/observation group), whereas relative risks for fatal outcomes due to recurrent venous thromboembolism or major bleeding are above the diagonal line (row defining placebo/observation group, column defining the experimental group). Significant results are presented in bold/light grey. Data for non-commercialized drugs (idraparinux, sulodexide, and ximelagatran) are provided in S5 Table. ASA: ASPIRIN; BID: TWICE DAILY; VKA: VITAMIN K ANTAGONIST.

## Discussion

The present network meta-analysis confirmed that extended anticoagulation significantly reduced the rate of recurrent VTE following an acute DVT or PE. This effect was mostly apparent for standard-dose VKA and DOAC, which were associated with a ≈80% and ≈75% relative risk reduction compared to placebo and aspirin, respectively. Consistently, individual pairwise comparisons confirmed that VKA and DOAC were more effective than ASA, whereas standard-dose VKA prevented more effectively recurrent VTE than low-dose VKA. DOAC also tended to be more effective than low-dose VKA. On the other hand, DOAC tended to be associated with a lower risk of MB. Consequently, standard-dose VKA and DOAC were associated with the highest net clinical benefit in the secondary prevention of VTE. Conversely, low-dose apixaban and rivaroxaban were not associated with differences in VTE recurrence, MB or net clinical benefit compared to full-dose DOAC.

Following the initial three months of anticoagulation for acute VTE, both physicians and patients face the important question of whether long-term anticoagulation should be maintained to prevent recurrent VTE. With the exception of patients with cancer and antiphospholipid syndrome, the risk for recurrent VTE after discontinuation of anticoagulation is mostly related to the characteristics of the index event, the recurrence rate averaging 2.5% and 4.5% per year after provoked and unprovoked VTE, respectively [24, 32]. This risk appears to be more important during the first year after anticoagulant discontinuation or in patients with recurrent episodes. Therefore, treatment for longer than 3 months is generally not recommended following a VTE provoked by a major transient risk factor, whereas indefinite anticoagulation is recommended for patients with recurrent events [4, 5]. However, the assessment of the risk of recurrence in patients with a first episode of unprovoked VTE is more complex. The risk of PE recurrence after a first unprovoked PE reaches 20% at 5 years [25, 33], with age and presence of antiphospholipid syndrome being associated with an increased risk of recurrence [34]. As a result, the American College of Chest Physician (ACCP) and European guidelines suggest that extended (no scheduled stop date) oral anticoagulation should be preconized [4] or considered [5] for patients with a first episode of unprovoked PE and low-to-moderate [4] or low [5] bleeding risk. Interestingly, while the ACCP guidelines suggests the use of DOAC over standard-adjusted dose VKA for extended treatment of VTE [4], the European guidelines rather suggest DOAC as an alternative option to VKA only [5]. This European recommendation is likely based on the fact that the risk of bleeding with DOAC, and particularly intracranial bleeding, was less than with VKA therapy in the acute management of VTE [35, 36] and atrial fibrillation [37, 38]. It is noteworthy, however, that limited direct or indirect comparisons were made between VKA and DOAC in the extended phase of anticoagulation for VTE.

In this regard, the present results add to the current literature by providing more specific estimates of VTE recurrence and MB with the diverse treatment strategies for the long-term management of VTE. Intriguingly, the present meta-analysis did not provide evidence of differences in efficacy and bleeding during extended anticoagulation between treatments, either as drug class (i.e. standard-dose VKA versus DOAC), dosage (standard- versus low-dose DOAC) or as individual agents, except for ASA that was associated with an increased risk of recurrence. These results differ from previous phase 3 trials comparing DOAC to low molecular weight heparins/VKA for the acute management of VTE that reported a decreased risk of bleeding associated with edoxaban, rivaroxaban and apixaban [39–42]. Whether a difference in patient characteristics may explain this discrepancy remains unknown. Indeed, while the absolute rates of MB in the included trials were similar to trials exploring acute treatment of VTE, the exclusion of patients with previous bleeding or recurrence in the acute phase of treatment may have led to a selection bias toward patients at lower risk of bleeding or recurrence. In addition, individual phase 3 trials that reported a reduction in bleeding risk with apixaban, edoxaban, and rivaroxaban generally included more than 4,000 patients and used a composite of MB and non-major clinically relevant bleeding [12, 13, 43], increasing the absolute number of events. The current meta-analysis may thus have lacked sufficient power to detect a potential difference in MB, reflected by the large confidence intervals of the estimates. However, the clinical relevance of non-major clinically relevant bleeding is still under debate [44].

More recently, the net clinical benefit of anticoagulation therapy, a composite endpoint defined as recurrent VTE or MB, was proposed as an attempt to fully capture the overall effects of anticoagulation in the secondary prevention of VTE [45]. The net clinical benefit is considered as a global appraisal of treatment effects, helping clinicians and patients weighting the advantages and the harms of therapy and personalizing the choice of treatment in a shared-decision process. Consistent with previous findings, the analysis of the net clinical benefit in our meta-analysis favored the use of DOAC or standard-adjusted dose VKA over ASA, low-dose VKA or observation alone (except for standard-dose rivaroxaban compared to low-dose VKA). Similarly, the frequentist network meta-analysis methods suggested that apixaban (2.5 and 5mg), and to a lesser extent dabigatran 150mg and rivaroxaban 10mg, were associated with the highest probability of being the best treatments in terms of net clinical benefit. It is noteworthy, however, that these results should be interpreted with great caution given that such analyses preclude the appropriate calculation of the confidence intervals of these probability estimates. In addition, despite the fact that VKA and DOAC were associated with a trend for reduced mortality, none of the antithrombotic drugs were associated with a reduction in fatal events, with the possible exception of apixaban versus ASA. It remains unknown if this result reflects a similar efficacy in preventing mortality or a lack of power due to the low number of recurrent fatal PE and fatal bleeding.

Contrary to two previous network meta-analyses [16, 46], the current study did not observe any significant difference in MB between VKA and the diverse DOAC. This discrepancy is likely related to the precision gained by the inclusion of 3 recent trials testing rivaroxaban 10 and 20 mg, standard dose VKA, and sulodexide [14, 25, 31]. Indeed, in the studies of Castelluci et al. and Sterne et al. [16, 46], the higher risk of MB with rivaroxaban was mainly derived from one trial [12] in which no MB occurred in the placebo arm whereas 4 (0.7%) occurred in the rivaroxaban one, yielding to statistically significant although uncertain increased risk of MB. Conversely, the effects of the diverse treatments on VTE recurrence were similar in both meta-analysis. Thus, with the inclusion of additional trials, as well as the description of the net clinical benefit of the diverse therapeutic strategies, the present meta-analysis likely provides a more precise estimate of the specific treatment effects of antithrombotic drugs for extended treatment of VTE.

Taken together, the present meta-analysis supports current guidelines suggesting that standard-dose VKA (INR 2 to 3) and DOAC are appropriate treatment strategies to prevent VTE recurrence. While apixaban, edoxaban, and rivaroxaban have a better safety profile over VKA for the acute management [39–42], DOAC were only associated with trends for reduced MB and their net clinical benefit were similar during extended anticoagulation compared to VKA. Pharmacoeconomic studies also suggested that apixaban, dabigatran and rivaroxaban were cost-effective alternatives to VKA for extended anticoagulation following acute VTE in Canada, United Kingdom, and United States of America [47–51], although these studies were funded by pharmaceutical companies. Ultimately, the choice of treatment for extended anticoagulation thus likely relies on patients’ preference and individual risk factors for adverse events with VKA and DOAC. Although considered as more convenient, DOAC are also associated with variable pharmacodynamic in case of specific medical conditions and drug-drug interactions [52]. In some situations, their efficacy and safety is still unknown [53]. Hence, our results may be reassuring for some patients well equilibrated under VKA for extended therapy.

Conversely, the place of ASA appears to be limited, being associated with a lower reduction of recurrent VTE compared to VKA or DOAC without a significant reduction in MB. ASA should thus be reserved to the minority of patients refusing to take or not tolerating any form of anticoagulants. It is noteworthy, however, that in the absence of direct effects on mortality, extended anticoagulation aims to mitigating the risk of non-fatal complications such as recurrent VTE, or post-thrombotic syndrome [11]. Unfortunately, the effects of anticoagulation on these outcomes were not reported in the trials. The decision on extended anticoagulation should therefore be based on the periodical re-assessment of risk/benefit ratio in a shared decision-making process, as currently recommended.

### Limitations of the study

There are several limitations to this study that should be considered. Firstly, the magnitude of treatment effect may be affected by the design of the included trials. Overall, the risk of bias was considered low, although length of follow-up that varied widely from one study to the other. However, this variation did not modify the treatment effect of VTE prevention using a Bayesian approach [16]. Secondly, characteristics of patients, such as index events (PE versus DVT), patient’s age, body mass index and comorbidities may modify the treatment effect of anticoagulation. In absence of patient level data, their influences cannot be explored. Thirdly, non-major clinically relevant bleeding were not taken into account and data about other relevant outcomes (myocardial infarction, stroke) were sparse or unavailable in the majority of trials, precluding specific analyses. Fourthly, patients recruited in most trials were not naïve to VKA. Patients at risk of bleeding could have been excluded, as the incidence of MB is highest in the initial months of treatment. Similarly, patients with a low time in therapeutic range were generally excluded, favouring the benefit of VKA. The design of randomized trials studying the extended anticoagulation may thus have overestimated the treatment effects of VKA [11]. Fifthly, the confidence intervals generated by the present meta-analysis were large despite the power gain related to network meta-analysis. It may be explained by the fact that the estimates were based on only one trial for several drugs. Thus, the absence of difference between treatment effects did not imply their equivalence. This pitfall was partially related to the low rate of events, especially for fatal events. Finally, most of the comparisons were only indirect and subject to artefacts caused by study designs, patient populations and other co-variables. These results should therefore be interpreted with extreme caution in the absence of head-to-head clinical trials. Network meta-analysis requires studies to be sufficiently similar in terms of treatment effect modifiers in order to verify the transitivity assumption to pool their results [54]. However, the frequentist approach and the relatively small number of included trials did not permit performing analysis according to covariables and to explore the transitivity assumption such as trial duration or patient characteristics. In the Bayesian meta-analysis of Castellucci et al.[16], durations of trials were explored by metaregression. The authors did not conclude a relation between duration of the trials and the treatment effect.

## Conclusion

In conclusion, standard dose VKA and DOAC shared similar effects on VTE recurrence and MB, whereas ASA and low-dose VKA were associated with the worst risk/benefit ratio. While DOAC were associated with a trend for reduced risk of MB compared to VKA, this effect remained non-significant during extended anticoagulant therapy. Conversely, low-dose apixaban and rivaroxaban were also associated with a similar risk of MB compared to standard-dose, as previously documented for low-dose VKA.

## Supporting information

S1 PRISMA Checklist (DOC)

S1 Text Data sources and searches. Medline and embase search strategies.
(DOCX)

S1 Table Number of events for each main outcome reported by 17 randomized trials for the secondary prevention of venous thromboembolic events. (DOCX)

S2 Table Study Quality. (DOCX)

S3 Table Sensitivity analysis describing the relative risk (95% confidence interval) from network meta-analysis for recurrent thromboembolism events and major bleeding for all pairwise comparisons including marketed and unmarketed (idraparinux, sulodexide, ximelagatran) drugs. (DOCX)

S4 Table Probability of being the best treatment according to the p-score computing using frequentist network meta-analysis for marketed and unmarketed drugs. (DOCX)

S5 Table Relative risk (95% confidence interval) from network meta-analysis for net clinical benefit and fatal recurrent venous thromboembolism and major bleeding for all pairwise comparisons for marketed and unmarketed drugs. (DOCX)

S6 Table Data allowing the calculation of estimates in network meta-analysis (format for netmeta package). (XLSX)
