# Supplementary material for: Extended anticoagulation for the secondary prevention of venous thromboembolic events: An updated network meta-analysis
Source: PLoS One. 2019 Apr 1;14(4):e0214134. doi: 10.1371/journal.pone.0214134 (PMC6443183; doi:10.1371/journal.pone.0214134)
Supplement: S1 Text — Medline and embase search strategies. (DOCX) [file pone.0214134.s002.docx]

**Methods**

**S1 Text – Data Sources and Searches – Medline and Embase search strategies**

MEDLINE search strategy:

("Pulmonary Embolism"[Mesh] OR "Venous Thrombosis"[Mesh] OR "Venous Thromboembolism"[Mesh]) AND ("Rivaroxaban"[Mesh] OR "Acenocoumarol"[Mesh] OR "Anticoagulants"[Mesh] OR "apixaban"[Supplementary Concept] OR "Factor Xa Inhibitors"[Mesh] OR "Dabigatran"[Mesh] OR "Aspirin"[Mesh] OR "edoxaban" [Supplementary Concept] OR "ximelagatran" [Supplementary Concept] OR acarboxyprothrombin [Supplementary Concept] OR "Rivaroxaban"[All Fields] OR "Acenocoumarol"[All Fields] OR "Anticoagulants"[All Fields] OR "apixaban"[Supplementary Concept] OR "Factor Xa Inhibitors"[All Fields] OR "Dabigatran"[All Fields] OR "Aspirin"[All Fields] OR "edoxaban" [All Fields] OR "ximelagatran" [All Fields]) AND ("Randomized Controlled Trial" [Publication Type] OR "Random Allocation"[Mesh] OR "Clinical Trials as Topic"[Mesh])

EMBASE search strategy:

('vein thrombosis'/exp OR 'venous thromboembolism'/exp) AND

('apixaban'/exp OR 'rivaroxaban'/exp OR 'dabigatran'/exp OR 'ximelagatran'/exp OR 'edoxaban'/exp OR 'blood clotting factor 10a inhibitor'/exp OR 'anticoagulant agent'/exp OR 'acetylsalicylic acid'/exp OR 'thrombin inhibitor'/exp OR 'antithrombocytic agent'/exp) AND ('randomized controlled trial'/exp OR 'controlled clinical trial'/exp OR 'randomization'/exp) AND [embase]/lim
