# Supplementary material for: Extended anticoagulation for the secondary prevention of venous thromboembolic events: An updated network meta-analysis
Source: PLoS One. 2019 Apr 1;14(4):e0214134. doi: 10.1371/journal.pone.0214134 (PMC6443183; doi:10.1371/journal.pone.0214134)
Supplement: S1 Table — (DOCX) [file pone.0214134.s003.docx]

**Results**

**S1 Table – Number of events for main outcomes reported by 17 randomized trials for the secondary prevention of venous thromboembolic events**

| Study (Patients) | Intervention groups | Number of recurrent VTE (%) | Number of recurrent VTE within the unprovoked VTE at baseline (%) | Fatal recurrent VTE (%) | Major bleeding events (%) | Fatal bleeding events (%) | Number of fatal events of all causes |
| --- | --- | --- | --- | --- | --- | --- | --- |
| LAFIT [1] (all VTE) | Placebo | 17 (20.5) | 17 (20.5) | 1 | 0 | 0 | 3 |
|  | VKA  (INR 2.0-3.0) | 1 (1.3) | 1 (1.3) | 0 | 3 | 0 | 1 |
| WODIT DVT [2] (DVT only) | Observation | 11 (8.3) | 11 (8.3) | 0 | 2 | 2 | 7 |
|  | VKA  (INR 2.0-3.0) | 4^@^ (3.0) | 4^@^ (3.0) | 0 | 4 | 0 | 7 |
| WODIT PE [3] (PE only) | Observation | NR^$^ (NR) | NR^$^ (NR) | 0^⌘^ | 1 | 0 | 7 |
|  | VKA  (INR 2.0-3.0) | NR^$^ (NR) | NR^$^ (NR) | 2^⌘^ | 3 | 0 | 12 |
| PADIS-PE [4] (PE only) | Placebo | 25 (13.4) | 25 (13.4) | 0 | 1 | 0 | 2 |
|  | VKA  (INR 2.0-3.0) | 3^⌃^ (1.6) | 3^⌃^ (1.6) | 0 | 4^⍺^ | 0 | 2 |
| AUREC-FVIII [5] (all VTE)^✻^ | Observation | 2 (11.8) | 2 (11.8) | NR | 0 | 0 | NR |
|  | VKA  (INR 2.0-3.0) | 2 (11.8) | 2 (11.8) | NR | 1 | 0 | NR |
| PROLONG [6] (all VTE)^⊂^ | Observation | 18 (14.8) | 18 (14.8) | 0 | 0 | 0 | 1 |
|  | VKA  (INR 2.0-3.0) | 2^∪^ (1.9) | 2^∪^  (1.9) | 0 | 1 | 0 | 1 |
| ELATE [7] (all VTE) | VKA  (INR 2.0-3.0) | 6^⍬^ (1.6) | 6^⍬^ (1.6) | 2^⍲^ | 8 | 0 | 8 |
|  | VKA  (INR 1.5-1.9) | 16^⍱^ (4.3) | 16^⍱^ (4.3) | 1^⍜^ | 9 | 0 | 16 |
| PREVENT [8] (all VTE) | Placebo | 37^⍤^ (14.6) | 37^⍤^ (14.6) | 2 | 2 | 1 | 8 |
|  | VKA  (INR 1.5-2.0) | 14^⎍^ (5.5) | 14^⎍^ (5.5) | 0 | 5 | 0 | 4 |
| Thrive III [9] (all VTE) | Placebo | 71 (11.5) | NR (NR) | 3 | 5 | 0 | 7 |
|  | Ximelagatran 24mg twice daily | 12 (1.9) | NR (NR) | 0 | 6 | 0 | 6 |
| RESONATE [10] (all VTE) | Placebo | 37 (5.5) | NR (NR) | 2^⌿^ | 0 | 0 | 2 |
|  | Dabigatran 150mg twice daily | 3 (0.4) | NR (NR) | 0 | 2 | 0 | 0 |
| REMEDY [10] (all VTE) | VKA  (INR 2.0-3.0) | 18 (1.3) | NR (NR) | 1 | 25 | 1 | 19 |
|  | Dabigatran 150mg twice daily | 26 (1.8) | NR (NR) | 1 | 13 | 0 | 17 |
| EINSTEIN-EXT [11] (all VTE) | Placebo | 42 (7.1) | NR (NR) | 1 | 0 | 0 | 2 |
|  | Rivaroxaban 20mg once daily | 8 (1.3) | NR (NR) | 0 | 4 | 0 | 1 |
| EINSTEIN-CHOICE [12] (all VTE) | ASA 100mg daily | 50 (4.4) | 26 (5.6) | 2 | 3 | 1 | 7 |
|  | Rivaroxaban 10mg once daily | 13 (1.1) | 7 (1.5) | 0 | 5 | 0 | 2 |
|  | Rivaroxaban 20mg once daily | 17 (1.5) | 8 (1.8) | 2 | 6 | 1 | 8 |
| AMPLIFIY-EXT [13] (all VTE) | Placebo | 73 (8.8) | NR (NR) | 7^⍃^ | 4 | 0 | 14 |
|  | Apixaban 5mg twice daily | 14 (1.7) | NR (NR) | 3^⍃^ | 1 | 0 | 4 |
|  | Apixaban 2.5mg twice daily | 14 (1.7) | NR (NR) | 2^⍃^ | 2 | 0 | 7 |
| Van Gogh [14]  (all VTE) | Placebo | 23 (3.7) | NR (NR) | 1 | 0 | 0 | 4 |
|  | Idraparinux 2.5mg s/c weekly | 6 (1.0) | NR (NR) | 2 | 11 | 3 | 9 |
| WARFASA [15] (all VTE) | Placebo | 43^⍀^ (21.7) | 43^⍀^ (21.7) | 1 | 1 | NR | 5 |
|  | ASA 100mg daily | 28^⍀^ (13.7) | 28^⍀^ (13.7) | 1 | 1 | NR | 6 |
| ASPIRE [16] (all VTE) | Placebo | 73 (17.8) | 73 (17.8) | 1 | 6 | 2 | 18 |
|  | ASA 100mg daily | 57 (13.9) | 57 (13.9) | 1 | 8 | 0 | 16 |
| SURVET [17] (all VTE) | Placebo | 30 (9.7) | 30 (9.7) | NR^⍴^ | 0 | 0 | 3 |
|  | Sulodexide 500 lipasemic units twice daily | 15 (4.9) | 15 (4.9) | NR^⍴^ | 0 | 0 | 1 |

VTE: venous thromboembolism; VKA: vitamin K antagonist; INR: international normalized ratio; NR: not reported; DVT: deep venous thromboembolism; PE: pulmonary embolism; ^@^3 of the 4 patients discontinued the medication before the end of the treatment period; ^$^Only one recurrence during the study treatment period, but not specified in which group; ^⌘^2 fatal VTE recurrences in the extended anticoagulation group after treatment discontinuation; ^⌃^After warfarin discontinuation; ^⍺^One had discontinued warfarin; ^⍹^During the entire follow-up; **^✻^**With FVIII levels >230 IU/dL; ^⊂^Abnormal d-dimer level 1 month after discontinuation of anticoagulation (received at least 3 months of VKA as initial treatment); ^∪^ 1 had stopped anticoagulation; ^λ^1 had a stroke, not specified in which group; ^Φ^2 had ischemic heart disease, 1 of whom died and was specified in the VKA group, whereas the other was not specified in which group; ^⍬^3 discontinued the study treatment and 2 had an INR under 2.0; ^⍱^5 discontinued the study treatment; ^⍲^1 had a possible PE while a myocardial infarction was also suspected; ^⍜^Fatal PE was strongly suspected; ^⍧^One death suspected; ^⍤^8 discontinued the treatment; ^⎍^7 discontinued the treatment; ^⍦^Ischemic stroke that became hemorrhagic; ^⌿^2 fatal recurrences not proven and one was not taking the treatment anymore; ^⌗^3 in VKA group and 1 dabigatran group had cardiovascular events that happened within 30 days after treatment was stopped; ^⍃^Deaths where PE could not be ruled out; ^⍀^39 in placebo group and 23 in ASA group had recurrent events while taking the study drug; ^Ω^ After the drug was discontinued; ^⍵^8 in placebo group and 4 in ASA group presented cardiovascular mortality, not further specified; ^⍴^One fatal recurrent VTE event, not further specified in which group;

**References**

1. Kearon C, Gent M, Hirsh J, Weitz J, Kovacs MJ, Anderson DR, et al. A comparison of three months of anticoagulation with extended anticoagulation for a first episode of idiopathic venous thromboembolism. N Engl J Med. 1999;340(12):901-7. Epub 1999/03/25. doi: 10.1056/NEJM199903253401201. PubMed PMID: 10089183.

2. Agnelli G, Prandoni P, Santamaria MG, Bagatella P, Iorio A, Bazzan M, et al. Three months versus one year of oral anticoagulant therapy for idiopathic deep venous thrombosis. Warfarin Optimal Duration Italian Trial Investigators. N Engl J Med. 2001;345(3):165-9. Epub 2001/07/21. doi: 10.1056/NEJM200107193450302. PubMed PMID: 11463010.

3. Agnelli G, Prandoni P, Becattini C, Silingardi M, Taliani MR, Miccio M, et al. Extended oral anticoagulant therapy after a first episode of pulmonary embolism. Ann Intern Med. 2003;139(1):19-25. Epub 2003/07/02. PubMed PMID: 12834314.

4. Couturaud F, Sanchez O, Pernod G, Mismetti P, Jego P, Duhamel E, et al. Six Months vs Extended Oral Anticoagulation After a First Episode of Pulmonary Embolism: The PADIS-PE Randomized Clinical Trial. JAMA. 2015;314(1):31-40. doi: 10.1001/jama.2015.7046.

5. Eischer L, Gartner V, Schulman S, Kyrle PA, Eichinger S, investigators A-F. 6 versus 30 months anticoagulation for recurrent venous thrombosis in patients with high factor VIII. Ann Hematol. 2009;88(5):485-90. Epub 2008/10/22. doi: 10.1007/s00277-008-0626-1. PubMed PMID: 18931845.

6. Palareti G, Cosmi B, Legnani C, Tosetto A, Brusi C, Iorio A, et al. D-dimer testing to determine the duration of anticoagulation therapy. N Engl J Med. 2006;355(17):1780-9. Epub 2006/10/27. doi: 10.1056/NEJMoa054444. PubMed PMID: 17065639.

7. Kearon C, Ginsberg JS, Kovacs MJ, Anderson DR, Wells P, Julian JA, et al. Comparison of low-intensity warfarin therapy with conventional-intensity warfarin therapy for long-term prevention of recurrent venous thromboembolism. N Engl J Med. 2003;349(7):631-9. Epub 2003/08/15. doi: 10.1056/NEJMoa035422. PubMed PMID: 12917299.

8. Ridker PM, Goldhaber SZ, Danielson E, Rosenberg Y, Eby CS, Deitcher SR, et al. Long-term, low-intensity warfarin therapy for the prevention of recurrent venous thromboembolism. N Engl J Med. 2003;348(15):1425-34. Epub 2003/02/26. doi: 10.1056/NEJMoa035029. PubMed PMID: 12601075.

9. Schulman S, Wahlander K, Lundstrom T, Clason SB, Eriksson H, Investigators TI. Secondary prevention of venous thromboembolism with the oral direct thrombin inhibitor ximelagatran. N Engl J Med. 2003;349(18):1713-21. Epub 2003/10/31. doi: 10.1056/NEJMoa030104. PubMed PMID: 14585939.

10. Schulman S, Kearon C, Kakkar AK, Schellong S, Eriksson H, Baanstra D, et al. Extended use of dabigatran, warfarin, or placebo in venous thromboembolism. N Engl J Med. 2013;368(8):709-18. Epub 2013/02/22. doi: 10.1056/NEJMoa1113697. PubMed PMID: 23425163.

11. Investigators E, Bauersachs R, Berkowitz SD, Brenner B, Buller HR, Decousus H, et al. Oral rivaroxaban for symptomatic venous thromboembolism. N Engl J Med. 2010;363(26):2499-510. Epub 2010/12/07. doi: 10.1056/NEJMoa1007903. PubMed PMID: 21128814.

12. Weitz JI, Lensing AWA, Prins MH, Bauersachs R, Beyer-Westendorf J, Bounameaux H, et al. Rivaroxaban or Aspirin for Extended Treatment of Venous Thromboembolism. N Engl J Med. 2017;376(13):1211-22. doi: 10.056/NEJMoa1700518. Epub 2017 Mar 18.

13. Agnelli G, Buller HR, Cohen A, Curto M, Gallus AS, Johnson M, et al. Apixaban for extended treatment of venous thromboembolism. N Engl J Med. 2013;368(8):699-708. Epub 2012/12/12. doi: 10.1056/NEJMoa1207541. PubMed PMID: 23216615.

14. van Gogh I, Buller HR, Cohen AT, Davidson B, Decousus H, Gallus AS, et al. Extended prophylaxis of venous thromboembolism with idraparinux. N Engl J Med. 2007;357(11):1105-12. Epub 2007/09/15. doi: 10.1056/NEJMoa067703. PubMed PMID: 17855671.

15. Becattini C, Agnelli G, Schenone A, Eichinger S, Bucherini E, Silingardi M, et al. Aspirin for preventing the recurrence of venous thromboembolism. N Engl J Med. 2012;366(21):1959-67. Epub 2012/05/25. doi: 10.1056/NEJMoa1114238. PubMed PMID: 22621626.

16. Brighton TA, Eikelboom JW, Mann K, Mister R, Gallus A, Ockelford P, et al. Low-dose aspirin for preventing recurrent venous thromboembolism. N Engl J Med. 2012;367(21):1979-87. Epub 2012/11/06. doi: 10.1056/NEJMoa1210384. PubMed PMID: 23121403.

17. Andreozzi GM, Bignamini AA, Davi G, Palareti G, Matuska J, Holy M, et al. Sulodexide for the Prevention of Recurrent Venous Thromboembolism: The Sulodexide in Secondary Prevention of Recurrent Deep Vein Thrombosis (SURVET) Study: A Multicenter, Randomized, Double-Blind, Placebo-Controlled Trial. Circulation. 2015;132(20):1891-7. doi: 10.161/CIRCULATIONAHA.115.016930. Epub 2015 Sep 25.
