# Supplementary material for: Extended anticoagulation for the secondary prevention of venous thromboembolic events: An updated network meta-analysis
Source: PLoS One. 2019 Apr 1;14(4):e0214134. doi: 10.1371/journal.pone.0214134 (PMC6443183; doi:10.1371/journal.pone.0214134)
Supplement: S2 Table — (DOCX) [file pone.0214134.s004.docx]

**S2 Table – Study Quality.**

| Study | Random sequence generation | Allocation concealment | Blinding | Incomplete data outcome | Selective reporting | Other bias |
| --- | --- | --- | --- | --- | --- | --- |
| **Standard-dose VKA (INR 2.0-3.0)** | | | | | | |
| Kearon 1999[1] | Patients were randomized, stratified by index event and medical center, randomly determined by block of two or four within each stratum by a computer algorithm.  (Low risk) | Randomization by a computer algorithm.  Sham INR.  (Low risk) | Double-blind and independent blinded outcome assessment.  Central adjudication.  (Low risk) | Description of withdrawals. Significant difference in between eligible and randomized patients (204 vs 162). No loss to follow-up.  (Low risk) | All outcomes are reported.  (Low risk) | Early termination of the trial after 162 patients enrolled because of the unequivocal reduction in the rate of recurrent VTE in the warfarin group (pre-specified).  (Unclear risk) |
| Agnelli 2001[2] | Patients were randomized, not further specified.  (Low risk) | Central randomization.  (Low risk) | Open label. Blinded independent assessment of the outcome event.  Central adjudication.  (Low risk) | Description of withdrawals.  No losses to follow-up.  (Low risk) | All outcomes are reported.  (Low risk) | No.  (Low risk) |
| Agnelli 2003[3] | Patients were randomized centrally in permuted block of six.  (Low risk) | Central randomization.  (Low risk) | Open label.  Blinded independent assessment of the outcome events.  Central adjudication.  (Low risk) | Description of withdrawals.  No losses to follow-up.  (Low risk) | All outcomes are reported.  (Low risk) | No.  (Low risk) |
| Couturaud 2015[4] | Patients were randomized, stratified by center, assigned in randomly permuted blocks of four or six by a computer algorithm.  (Low risk) | Central computerized Internet-based system.  (Low risk) | Double-blind. Outcomes were reviewed by a blinded independent centralized addiction committee.  (Low risk) | Description of withdrawals and method of imputation for missing data.  (Low risk) | All outcomes are reported.  (Low risk) | Early termination of the trial because sufficient data to end the follow-up after 24 months.  (Unclear risk) |
| Eischer 2009[5] | Patients were randomized, not further specified.  (Unclear risk) | (Unclear risk) | Open label.  Adjudicators were blinded and unaware of allocation. (Cochrane contacted the authors)  (Unclear risk) | Description of withdrawals.  No losses to follow-up.  (Low risk) | All outcomes are reported.  (Low risk) | No.  (Low risk) |
| Palareti 2006[6] | Patients were randomized, with a different randomization sequence for each study site, in a block size of 10.  (Low risk) | A different randomization sequence for each study site was generated by a computer and encapsulated in a randomization program.  (Low risk) | Open label.  Blinded assessment of the outcome event.  Central adjudication.  (Unclear risk) | Description of withdrawals.  (Low risk) | All outcomes are reported.  (Low risk) | No.  (Low risk) |
| **Low-dose VKA (INR 1.5-2.0)** | | | | | | |
| Kearon 2003[7] | Patients were randomized, stratified by clinical center and length of initial anticoagulation, randomly determined in blocks of two or four by a computer algorithm.  (Low risk) | Central allocation.  Sham INR.  (Low risk) | Double-blind.  Blinded independent assessment of the outcome event.  (Low risk) | Description of withdrawals.  (Low risk) | All outcomes are reported.  (Low risk) | No.  (Low risk) |
| Ridker 2003[8] | Patients were randomized, stratified by clinical site, time since index event (<6 months or >6 months) and whether or not the index event was the patient’s first venous thromboembolism.  (Low risk) | Central randomization.  Sham INR.  (Low risk) | Double-blind. Blinded independent assessment of the outcome event.  (Low risk) | Description of withdrawals.  (Low risk) | All outcomes are reported.  (Low risk) | Early termination of the trial because there was strong evidence of efficacy and the monitoring boundary specified by the Lan-DeMets procedure has been crossed.  (Low risk) |
| **Direct thrombin inhibitors** | | | | | | |
| Schulman 2003[9] | Patients were randomized, stratified according to the presence or absence of active cancer during the previous five years by a computer-generated randomization list.  (Low risk) | Computer generated randomization list. Allocation concealed.  (Low risk) | Double-blind.  Blinded independent assessment of the outcome event.  Central adjudication.  (Low risk) | Description of withdrawals and method of imputation for missing data.  (Low risk) | All outcomes are reported.  (Low risk) | No.  (Low risk) |
| Schulman 2013[10] | Patients were randomized, stratified according to presence of absence of cancer and index event, by an interactive voice-response system.  (Low risk) | Central randomization.  (Low risk) | Double-blind.  Blinded assessment of the outcome event.  Central adjudication.  (Low risk) | Description of withdrawals and method of imputation for missing data.  (Low risk) | All outcomes are reported.  (Low risk) | No.  (Low risk) |
| Schulman 2013[10] | Patients were randomized, stratified according to presence of absence of cancer and index event, by an interactive voice-response system.  (Low risk) | Central randomization.  Sham INR.  (Low) | Double-blind.  Blinded assessment of the outcome event.  Central adjudication.  (Low risk) | Description of withdrawals and method of imputation for missing data.  (Low risk) | All outcomes are reported.  (Low risk) | No.  (Low risk) |
| **Factor Xa inhibitors** | | | | | | |
| Einstein Investigators 2010[11] | Patients were randomized, stratified by country.  (Low risk) | Central allocation by interactive voice response system.  (Low risk) | Double-blind.  Blinded independent assessment of the outcome event.  Central adjudication.  (Low risk) | Description of withdrawals and method of imputation for missing data.  (Low risk) | All outcomes are reported.  (Low risk) | No.  (Low risk) |
| Weitz 2017[12] | Patients were randomized with block size of six, stratified according to index diagnosis and country.  (Low risk) | Central allocation by an interactive voice or web response system.  (Low risk) | Double-blind.  Blinded independent assessment of the outcome event.  Central adjudication.  (Low risk) | Description of withdrawals and method of imputation for missing data.  (Low risk) | All outcomes are reported.  (Low risk) | No.  (Low risk) |
| Agnelli 2013[13] | Patients were randomized, stratified according to index diagnosis and previous participation to AMPLIFY trial.  (Low risk) | Central allocation by an interactive voice system.  (Low risk) | Double-blind.  Blinded independent assessment of the outcome event.  Central adjudication.  (Low risk) | Description of withdrawals and method of imputation for missing data.  (Low risk) | All outcomes are reported.  (Low risk) | No.  (Low risk) |
| Van Gogh 2007[14] | Patients were randomized, stratified according to the study center and the anticoagulant received in the 6 months before entering randomization, by a computerized voice-response system.  (Low risk) | Central randomization by a computerized voice-response system.  (Low risk) | Double-blind.  Blinded independent assessment of the outcome event.  Central adjudication.  (Low risk) | Description of withdrawals and method of imputation for missing data.  (Low risk) | All outcomes are reported.  (Low risk) | No.  (Low risk) |
| **Low-dose ASA** | | | | | | |
| Becattini 2012[15] | Patients were randomized, not further specified.  (Low risk) | Allocation concealed.  (Low risk) | Double-blind.  Blinded independent assessment of the outcome event.  (Low risk) | Description of withdrawals and method of imputation for missing data.  (Low risk) | All outcomes are reported.  (Low risk) | No.  (Low risk) |
| Brighton 2012[16] | Patients were randomized, stratified according to center and duration of initial anticoagulation.  (Low risk) | Central allocation by a Web based randomization system.  (Low risk) | Double-blind.  Blinded independent assessment of the outcome event.  (Low risk) | Description of withdrawals and method of imputation for missing data.  (Low risk) | All outcomes are reported.  (Low risk) | No.  (Low risk) |
| **Glycosaminoglycans** | | | | | | |
| Andreozzi 2015[17] | Patients were randomized based on a computer-generated randomization lists in block of four by an independent operating unit.  (Low risk) | Central randomization by an independent committee.  (Low risk) | Double-blind.  Blinded assessment of the outcome event.  Central adjudication.  (Low risk) | Description of withdrawals and method of imputation for missing data.  (Low risk) | All outcomes are reported.  (Low risk) | No.  (Low risk) |

ASA: aspirin; VTE: venous thromboembolism.

**References**

1. Kearon C, Gent M, Hirsh J, Weitz J, Kovacs MJ, Anderson DR, et al. A comparison of three months of anticoagulation with extended anticoagulation for a first episode of idiopathic venous thromboembolism. N Engl J Med. 1999;340(12):901-7. Epub 1999/03/25. doi: 10.1056/nejm199903253401201. PubMed PMID: 10089183.

2. Agnelli G, Prandoni P, Santamaria MG, Bagatella P, Iorio A, Bazzan M, et al. Three months versus one year of oral anticoagulant therapy for idiopathic deep venous thrombosis. Warfarin Optimal Duration Italian Trial Investigators. N Engl J Med. 2001;345(3):165-9. Epub 2001/07/21. doi: 10.1056/nejm200107193450302. PubMed PMID: 11463010.

3. Agnelli G, Prandoni P, Becattini C, et al. EXtended oral anticoagulant therapy after a first episode of pulmonary embolism. Annals of Internal Medicine. 2003;139(1):19-25. doi: 10.7326/0003-4819-139-1-200307010-00008.

4. Couturaud F, Sanchez O, Pernod G, Mismetti P, Jego P, Duhamel E, et al. Six Months vs Extended Oral Anticoagulation After a First Episode of Pulmonary Embolism: The PADIS-PE Randomized Clinical Trial. Jama. 2015;314(1):31-40. Epub 2015/07/08. doi: 10.1001/jama.2015.7046. PubMed PMID: 26151264.

5. Eischer L, Gartner V, Schulman S, Kyrle PA, Eichinger S. 6 versus 30 months anticoagulation for recurrent venous thrombosis in patients with high factor VIII. Ann Hematol. 2009;88(5):485-90. Epub 2008/10/22. doi: 10.1007/s00277-008-0626-1. PubMed PMID: 18931845.

6. Palareti G, Cosmi B, Legnani C, Tosetto A, Brusi C, Iorio A, et al. D-dimer testing to determine the duration of anticoagulation therapy. N Engl J Med. 2006;355(17):1780-9. Epub 2006/10/27. doi: 10.1056/NEJMoa054444. PubMed PMID: 17065639.

7. Kearon C, Ginsberg JS, Kovacs MJ, Anderson DR, Wells P, Julian JA, et al. Comparison of low-intensity warfarin therapy with conventional-intensity warfarin therapy for long-term prevention of recurrent venous thromboembolism. N Engl J Med. 2003;349(7):631-9. Epub 2003/08/15. doi: 10.1056/NEJMoa035422. PubMed PMID: 12917299.

8. Ridker PM, Goldhaber SZ, Danielson E, Rosenberg Y, Eby CS, Deitcher SR, et al. Long-term, low-intensity warfarin therapy for the prevention of recurrent venous thromboembolism. N Engl J Med. 2003;348(15):1425-34. Epub 2003/02/26. doi: 10.1056/NEJMoa035029. PubMed PMID: 12601075.

9. Schulman S, Wahlander K, Lundstrom T, Clason SB, Eriksson H. Secondary prevention of venous thromboembolism with the oral direct thrombin inhibitor ximelagatran. N Engl J Med. 2003;349(18):1713-21. Epub 2003/10/31. doi: 10.1056/NEJMoa030104. PubMed PMID: 14585939.

10. Schulman S. Extended anticoagulation in venous thromboembolism. N Engl J Med. 2013;368(24):2329. Epub 2013/06/14. doi: 10.1056/NEJMc1304815. PubMed PMID: 23758240.

11. Investigators E, Bauersachs R, Berkowitz SD, Brenner B, Buller HR, Decousus H, et al. Oral rivaroxaban for symptomatic venous thromboembolism. N Engl J Med. 2010;363(26):2499-510. Epub 2010/12/07. doi: 10.1056/NEJMoa1007903. PubMed PMID: 21128814.

12. Weitz JI, Lensing AWA, Prins MH, Bauersachs R, Beyer-Westendorf J, Bounameaux H, et al. Rivaroxaban or Aspirin for Extended Treatment of Venous Thromboembolism. N Engl J Med. 2017;376(13):1211-22. Epub 2017/03/21. doi: 10.1056/NEJMoa1700518. PubMed PMID: 28316279.

13. Agnelli G, Buller HR, Cohen A, Curto M, Gallus AS, Johnson M, et al. Apixaban for extended treatment of venous thromboembolism. N Engl J Med. 2013;368(8):699-708. Epub 2012/12/12. doi: 10.1056/NEJMoa1207541. PubMed PMID: 23216615.

14. van Gogh I, Buller HR, Cohen AT, Davidson B, Decousus H, Gallus AS, et al. Extended prophylaxis of venous thromboembolism with idraparinux. N Engl J Med. 2007;357(11):1105-12. Epub 2007/09/15. doi: 10.1056/NEJMoa067703. PubMed PMID: 17855671.

15. Becattini C, Agnelli G, Schenone A, Eichinger S, Bucherini E, Silingardi M, et al. Aspirin for preventing the recurrence of venous thromboembolism. N Engl J Med. 2012;366(21):1959-67. Epub 2012/05/25. doi: 10.1056/NEJMoa1114238. PubMed PMID: 22621626.

16. Brighton TA, Eikelboom JW, Mann K, Mister R, Gallus A, Ockelford P, et al. Low-dose aspirin for preventing recurrent venous thromboembolism. N Engl J Med. 2012;367(21):1979-87. Epub 2012/11/06. doi: 10.1056/NEJMoa1210384. PubMed PMID: 23121403.

17. Andreozzi GM, Bignamini AA, Davi G, Palareti G, Matuska J, Holy M, et al. Sulodexide for the Prevention of Recurrent Venous Thromboembolism: The Sulodexide in Secondary Prevention of Recurrent Deep Vein Thrombosis (SURVET) Study: A Multicenter, Randomized, Double-Blind, Placebo-Controlled Trial. Circulation. 2015;132(20):1891-7. Epub 2015/09/27. doi: 10.1161/circulationaha.115.016930. PubMed PMID: 26408273; PubMed Central PMCID: PMCPMC4643750.
