# Supplementary material for: Extended anticoagulation for the secondary prevention of venous thromboembolic events: An updated network meta-analysis
Source: PLoS One. 2019 Apr 1;14(4):e0214134. doi: 10.1371/journal.pone.0214134 (PMC6443183; doi:10.1371/journal.pone.0214134)
Supplement: S3 Table — (DOCX) [file pone.0214134.s005.docx]

**S3 Table – Sensitivity analysis describing the relative risk (95% confidence interval) from network meta-analysis for recurrent thromboembolism events and major bleeding for all pairwise comparisons including marketed and unmarketed (idraparinux, sulodexide, ximelagatran) drugs.**

| **Placebo or  observation** | 0.61  (0.11-3.31) | **3.13**  **(1.37-7.16)** | **3.23 (1.16-8.99)** | 1.71 (0.61-4.75) | 0.50 (0.09-2.72) | 0.25 (0.03-2.27) | 1.29  (0.17-9.78) | 1.77  (0.27-11.44) | **24.04**  **(1.42-407-12**) | -* | 1.20  (0.37-3.90) |
| --- | --- | --- | --- | --- | --- | --- | --- | --- | --- | --- | --- |
| **0.71 (0.55-0.91)** | **ASA** | 5.16  (0.78-34.04) | 5.32  (0.73-38.56) | 2.81  (0.39-20.39) | 0.82  (0.07-9.06) | 0.42  (0.03-6.68) | 2.13  (0.53-8.53) | 2.92  (0.81-10.59) | **39.63**  **(1.46-1072.94)** | -* | 1.97  (0.25-15.60) |
| **0.16 (0.10-0.26)** | **0.22 (0.13-0.39)** | **VKA**  **(INR 2.0-3.0)** | 1.03  (0.44-2.39) | 0.55  (0.28-1.05) | 0.16  (0.02-1.05) | **0.08**  **(0.01-0.84)** | 0.41  (0.05-3.68) | 0.57  (0.07-4.35) | 7.86  (0.40-146.41) | -* | 0.38  (0.09-1.62) |
| **0.39 (0.23-0.65)** | **0.55 (0.31-0.97)** | **2.47 (1.34-4.55)** | **VKA**  **(INR 1.5-2.0)** | 0.53  (0.18-1.52) | 0.15  (0.02-1.12) | **0.08**  **(0.01-0.88)** | 0.40  (0.04-3.87) | 0.55  (0.07-4.61) | 7.45  (0.37-151.05) | -* | **0.37**  **(0.11-0.86)** |
| **0.18 (0.09-0.35)** | **0.26 (0.13-0.52)** | 1.16 (0.68-1.99) | 0.47 (0.22-1.02) | **Dabigatran  150 mg BID** | 0.29 (0.04-2.12) | 0.15 (0.01-1.67) | 0.76  (0.08-7.31) | 1.04  (0.12-8.71) | 14.08  (0.70-285.23) | -* | 0.70  (0.15-3.35) |
| **0.19 (0.11-0.34)** | **0.27 (0.15-0.50)** | 1.22 (0.57-2.60) | 0.49 (0.23-1.06) | 1.03 (0.43-2.49) | **Apixaban**  **2.5 mg BID** | 0.51 (0.05-5.60) | 2.58  (0.18-36.21) | 3.54  (0.28-44.05) | 48.09  (1.78-1301.09) | -* | 2.40  (0.30-18.91) |
| **0.20 (0.11-0.34)** | **0.28 (0.15-0.51)** | 1.24 (0.58-2.64) | 0.50 (0.23-1.08) | 1.05 (0.43-2.54) | 1.02 (0.49-2.12) | **Apixaban 5 mg BID** | 5.08  (0.26-100.17) | 6.97  (0.39-123.63) | **94.55**  **(2.64-3382.71)** | -* | 4.71  (0.39-56.69) |
| **0.18 (0.09-0.35)** | **0.25 (0.14-0.46)** | 1.15 (0.51-2.60) | 0.47 (0.20-1.06) | 0.97 (0.38-2.47) | 0.94 (0.40-2.21) | 0.93 (0.39-2.18) | **Rivaroxaban  10 mg daily** | 1.37  (0.42-4.43) | 18.62  (0.57-604.04) | -* | 0.93  (0.09-9.86) |
| **0.22 (0.14-0.36)** | **0.32 (0.20-0.49)** | 1.42 (0.72-2.83) | 0.58 (0.29-1.16) | 1.20 (0.53-2.75) | 1.17 (0.56-2.43) | 1.15 (0.55-2.39) | 1.24  (0.63-2.44) | **Rivaroxaban  20 mg daily** | 13.57  (0.46-402.29) | -* | 0.86  (0.07-6.16) |
| **0.27**  **(0.11-0.67)** | 0.40  (0.14-1.14) | 1.07  (0.49-2.35) | 0.70  (0.25-1.97) | 1.56  (0.45-5.39) | 1.42  (0.50-4.08) | 1.40  (0.49-4.01) | 1.51  (0.50-4.52) | 1.26  (0.40-3.96) | **Idraparinux**  **2.5 mg** | -* | 20.07  (0.94-430.62) |
| **0.50**  **(0.28-0.91)** | 0.73  (0.32-1.66) | **3.19**  **(1.46-6.98)** | 1.29  (0.59-2.86) | **2.87**  **(1.01-8.19)** | 2.62  (1.15-5.96) | 2.57  (1.13-5.85) | **2.77**  **(1.15-6.67)** | 2.31  (0.90-5.91) | 1.84  (0.63-5.38) | **Sulodexide** | -* |
| **0.17**  **(0.09-0.31)** | **0.24**  **(0.11-0.56)** | 1.07  (0.49-2.35) | 0.44  (0.20-0.96) | 0.97  (0.34-2.76) | 0.88  (0.39-2.01) | 0.87  (0.38-1.97) | 0.93  (0.39-2.25) | 0.78  (030-1.99) | 0.62  (0.21-1.81) | **0.34**  **(0.14-0.79)** | **Ximelagatran**  **24 mg** |

Relative risks for recurrent venous thromboembolism are below the diagonal line (row defining the experimental group, column defining the placebo/observation group), whereas relative risks for major bleeding are above the diagonal line (row defining placebo/observation group, column defining the experimental group). Significant results are represented in bold/light grey.

* Zero event in each arms. Estimate was not computable.
