# Supplementary material for: Extended anticoagulation for the secondary prevention of venous thromboembolic events: An updated network meta-analysis
Source: PLoS One. 2019 Apr 1;14(4):e0214134. doi: 10.1371/journal.pone.0214134 (PMC6443183; doi:10.1371/journal.pone.0214134)
Supplement: S4 Table — (DOCX) [file pone.0214134.s006.docx]

**S4 Table – Probability of being the best treatment according to the p-score computing using frequentist network meta-analysis for marketed and unmarketed drugs.**

| **Treatment** | **Recurrence  of VTE** | **Major  bleeding** | **Net clinical  benefit** | **Fatal recurrent VTE and MB** |
| --- | --- | --- | --- | --- |
| Placebo/observation | 0% | 77% | 2% | 34% |
| ASA 100 mg DIE | 13% | 75% | 18% | 15% |
| Low-dose VKA (INR 1.5-2.0) | 27% | 22% | 30% | 74% |
| Standard-dose VKA (INR 2.0-3.0) | 82% | 22% | 54% | 51% |
| Dabigatran 150mg BID | 69% | 45% | 73% | 72% |
| Apixaban 2.5 mg BID | 67% | 79% | 81% | 70% |
| Apixaban 5 mg BID | 66% | 89% | 84% | 59% |
| Rivaroxaban 10 mg DIE | 71% | 52% | 76% | 66% |
| Rivaroxaban 20 mg DIE | 55% | 41% | 61% | 25% |
| Idraparinux 2.5 mg | 48% | 4% | 14% | 7% |
| Sulodexide | 21% | -* | 31% | -* |
| Ximelagatran 24 mg | 76% | 56% | 31% | 78% |

^*^ Not estimable because of zero event in the two arms of trial assessing sulodexide
