# Supplementary material for: Extended anticoagulation for the secondary prevention of venous thromboembolic events: An updated network meta-analysis
Source: PLoS One. 2019 Apr 1;14(4):e0214134. doi: 10.1371/journal.pone.0214134 (PMC6443183; doi:10.1371/journal.pone.0214134)
Supplement: S5 Table — (DOCX) [file pone.0214134.s007.docx]

**S5 Table – Sensitivity analysis describing the relative risk (95% confidence interval) from network meta-analysis for net clinical benefit and fatal recurrent venous thromboembolism and major bleeding for all pairwise comparisons including marketed and unmarketed drugs.**

| **Placebo or  observation** | 0.61  (0.11-3.31) | **3.13**  **(1.37-7.16)** | **3.23 (1.16-8.99)** | 1.71 (0.61-4.75) | 0.50 (0.09-2.72) | 0.25 (0.03-2.27) | 1.29  (0.17-9.78) | 1.77  (0.27-11.44) | 5.23  (0.61-44.61) | -^*^ | 0.14  (0.01-2.76) |
| --- | --- | --- | --- | --- | --- | --- | --- | --- | --- | --- | --- |
| **0.71 (0.55-0.91)** | **ASA** | 5.16  (0.78-34.04) | 5.32  (0.73-38.56) | 2.81  (0.39-20.39) | 0.82  (0.07-9.06) | 0.42  (0.03-6.68) | 2.13  (0.53-8.53) | 2.92  (0.81-10.59) | 2.27  (0.19-26.79) | -^*^ | 0.06  (0.00-1.53) |
| **0.16 (0.10-0.26)** | **0.22 (0.13-0.39)** | **VKA**  **(INR 2.0-3.0)** | 1.03  (0.44-2.39) | 0.55  (0.28-1.05) | 0.16  (0.02-1.05) | **0.08**  **(0.01-0.84)** | 0.41  (0.05-3.68) | 0.57  (0.07-4.35) | 9.22  (0.68-125.26) | -^*^ | 0.25  (0.01-6.91) |
| **0.39 (0.23-0.65)** | **0.55 (0.31-0.97)** | **2.47 (1.34-4.55)** | **VKA**  **(INR 1.5-2.0)** | 0.53  (0.18-1.52) | 0.15  (0.02-1.12) | **0.08**  **(0.01-0.88)** | 0.40  (0.04-3.87) | 0.55  (0.07-4.61) | **24.27**  **(1.23-476.90)** | -^*^ | 0.38  (0.05-2.68) |
| **0.18 (0.09-0.35)** | **0.26 (0.13-0.52)** | 1.16 (0.68-1.99) | 0.47 (0.22-1.02) | **Dabigatran  150 mg BID** | 0.29 (0.04-2.12) | 0.15 (0.01-1.67) | 0.76  (0.08-7.31) | 1.04  (0.12-8.71) | **21.33**  **(1.07-426.70)** | -^*^ | 0.58  (0.02-21.86) |
| **0.19 (0.11-0.34)** | **0.27 (0.15-0.50)** | 1.22 (0.57-2.60) | 0.49 (0.23-1.06) | 1.03 (0.43-2.49) | **Apixaban**  **2.5 mg BID** | 0.51 (0.05-5.60) | 2.58  (0.18-36.21) | 3.54  (0.28-44.05) | **18.30**  **(1.28-260.66)** | -^*^ | 0.50  (0.02-14.24) |
| **0.20 (0.11-0.34)** | **0.28 (0.15-0.51)** | 1.24 (0.58-2.64) | 0.50 (0.23-1.08) | 1.05 (0.43-2.54) | 1.02 (0.49-2.12) | **Apixaban 5 mg BID** | 5.08  (0.26-100.17) | 6.97  (0.39-123.63) | 11.99  (0.95-151.01) | -^*^ | 0.33  (0.01-8.47) |
| **0.18 (0.09-0.35)** | **0.25 (0.14-0.46)** | 1.15 (0.51-2.60) | 0.47 (0.20-1.06) | 0.97 (0.38-2.47) | 0.94 (0.40-2.21) | 0.93 (0.39-2.18) | **Rivaroxaban  10 mg daily** | 1.37  (0.42-4.43) | 18.87  (0.42-851.22) | -^*^ | 0.51  (0.01-38.79) |
| **0.22 (0.14-0.36)** | **0.32 (0.20-0.49)** | 1.42 (0.72-2.83) | 0.58 (0.29-1.16) | 1.20 (0.53-2.75) | 1.17 (0.56-2.43) | 1.15 (0.55-2.39) | 1.24  (0.63-2.44) | **Rivaroxaban  20 mg daily** | 3.16  (0.21-48.07**)** | -^*^ | 0.09  (0.00-2.59) |
| 0.77  (0.42-1.43) | 1.13  (0.58-2.18) | **2.54**  **(1.24-5.21)** | 1.53  (0.72-3.23) | **3.19**  **(1.45-7.03)** | **3.72**  **(1.65-8.38**) | **3.90**  **(1.171-8.88)** | 3.33  (1.45-7.68) | 2.62  (1.25-5.50) | **Idraparinux**  **2.5 mg** | -^*^ | 0.03  (0.00-1.06) |
| **0.50**  **(0.28-0.91)** | 0.73  (0.38-1.39) | 2.65  (0.82-3.33) | 0.99  (0.47-2.07) | 2.07  (0.95-4.50) | **2.41**  **(1.08-5.37)** | **2.53**  **(1.13-5.69)** | 2.16  (0.95-4.93) | 1.70  (0.82-3.52) | 0.65  (0.27-1.53) | **Sulodexide** | -^*^ |
| **0.24**  **(0.14-0.39)** | 0.34  (0.20-0.60) | 0.78  (0.42-1.45) | **0.47**  **(0.24-0.90)** | 0.98  (0.48-1.97) | 1.14  (0.55-2.36) | 1.19  (0.57-2.50) | 1.02  (0.48-2.17) | 0.80  (0.42-1.53) | **0.31**  **(0.14-0.68)** | 0.47  (0.22-1.05) | **Ximelagatran**  **24 mg** |

Relative risks for net clinical benefit are below the diagonal line (row defining the experimental group, column defining the placebo/observation group), whereas relative risks for fatal outcomes due to recurrent venous thromboembolism or major bleeding are above the diagonal line (row defining placebo/observation group, column defining the experimental group). Significant results are presented in bold/light grey.

ASA: aspirin; BID: twice daily; VKA: vitamin K antagonist. * Zero event in each arms. Estimate was not computable.
